# Colicin-Mediated Transport of DNA through the Iron Transporter FepA

**DOI:** 10.1128/mBio.01787-21

**Published:** 2021-09-21

**Authors:** Ruth Cohen-Khait, Ameya Harmalkar, Phuong Pham, Melissa N. Webby, Nicholas G. Housden, Emma Elliston, Jonathan T. S. Hopper, Shabaz Mohammed, Carol V. Robinson, Jeffrey J. Gray, Colin Kleanthous

**Affiliations:** a Department of Biochemistry, University of Oxford, Oxford, United Kingdom; b Chemical & Biomolecular Engineering, Johns Hopkins University, Baltimore, Maryland, USA; c Department of Chemistry, University of Oxford, Oxford, United Kingdom; CEH-Oxford

**Keywords:** membrane transport, bacteriocins, antibiotic resistance, conformational changes, Rosetta flexible backbone, DNA delivery

## Abstract

Colicins are protein antibiotics deployed by Escherichia coli to eliminate competing strains. Colicins frequently exploit outer membrane (OM) nutrient transporters to penetrate the selectively permeable bacterial cell envelope. Here, by applying live-cell fluorescence imaging, we were able to monitor the entry of the pore-forming toxin colicin B (ColB) into E. coli and localize it within the periplasm. We further demonstrate that single-stranded DNA coupled to ColB can also be transported to the periplasm, emphasizing that the import routes of colicins can be exploited to carry large cargo molecules into bacteria. Moreover, we characterize the molecular mechanism of ColB association with its OM receptor FepA by applying a combination of photoactivated cross-linking, mass spectrometry, and structural modeling. We demonstrate that complex formation is coincident with large-scale conformational changes in the colicin. Thereafter, active transport of ColB through FepA involves the colicin taking the place of the N-terminal half of the plug domain that normally occludes this iron transporter.

## INTRODUCTION

Bacteria are the most common and diverse form of life on earth. The remarkable abundance of different bacterial strains and species capable of surviving in almost any environment frequently leads to competition for space and resources (1). Competition for scarce nutrients has led to the evolution of nutrient uptake systems, such as the secretion of siderophores to chelate bioavailable iron, with the iron-siderophore complex captured by high-affinity receptors and actively transported across the cell envelope (2). Competing bacteria also deploy weapons in the form of enzymes targeting either components of the cell wall or nucleic acids (3) or depolarizing pores that disrupt the electrochemical potential across the inner membrane (4). Elimination of competing bacteria while kin bacteria are unharmed is achieved through the coexpression of toxin-specific immunity proteins that render the toxin inactive within producing strains (5). Cytotoxic proteins can be delivered either in a contact-dependent manner, targeting neighboring cells relying on the assembly of supramolecular machineries (6), or through secretion into the milieu as exemplified by bacteriocins (7).

Colicins, the bacteriocins of E. coli, have been extensively studied, with over 20 different examples described (8). Once released, colicins breach the envelope of their target cell to elicit their cytotoxic activity (9). The cell envelope of Gram-negative bacteria is comprised of an asymmetric outer membrane (OM) with an outer leaflet comprised of lipopolysaccharide and a phospholipid inner leaflet, providing a robust layer of defense surrounding the energized inner membrane (IM) and the intervening periplasm (10). Colicins are large (29 to 75 kDa) proteins that cannot diffuse through the cell envelope of their target cell (11) and must find a route across the OM (12). Unlike the proton-motive force (PMF) of the IM, the OM is not directly energized, and energy-dependent processes at the OM such as protein import are coupled to the IM through *trans*-periplasmic complexes. The Tol-Pal system, composed of the TolQ-TolR-TolA complex in the IM, TolB in the periplasm, and Pal anchored to the inner leaflet of the OM, stabilizes the OM during cell division (13). The structurally related Ton system, composed of the TonB-ExbB-ExbD complex in the IM, powers active transport of nutrients such as siderophores through specialized TonB-dependent receptors in the OM (14). Both the Tol-Pal and the Ton systems are exploited by colicins to energize their translocation across the cell envelope.

Colicins typically contain three structural domains, a central receptor (R)-binding domain, which anchors the toxin to the cell surface, an N-terminal translocation (T) domain implicated in OM translocation via the Tol-Pal, or a Ton system of a C-terminal cytotoxic domain. Colicin B (ColB) is a pore-forming toxin that was one of the earliest colicins to be described (15). However, little is known about the cellular translocation process of ColB beyond its dependence on the OM ferric enterobactin transporter FepA and the Ton system (16). No additional OM proteins have been identified for ColB toxicity, which may explain why, unlike most other colicins, ColB is composed of only two functional domains: an N-terminal domain that serves as both a receptor-binding domain and a translocation domain (ColB-RT) and a pore-forming, C-terminal cytotoxic domain (17). The ColB receptor FepA is a 22-stranded β-barrel TonB-dependent transporter (TBDT) with an N-terminal plug domain blocking its lumen (18–20).

Here, we elucidate the mechanism by which ColB interacts with its receptor FepA and its active transport across the OM. Applying live-cell fluorescence microscopy, we visualize the translocation of the ColB-RT domain to the periplasm of E. coli, demonstrating that translocation requires FepA at the OM and depends on colicin’s TonB box. We applied a combined approach of *in vitro* and *in vivo* photoactivated cross-linking, mass spectrometry, and structural modeling with Rosetta to monitor the key stages in the ColB-FepA association process. We also demonstrate that the import route of ColB can be used to import large macromolecules, in this instance single-stranded DNA (ssDNA), into bacteria.

## RESULTS

### ColB can transport ssDNA into E. coli via FepA.

**FIG 1:**
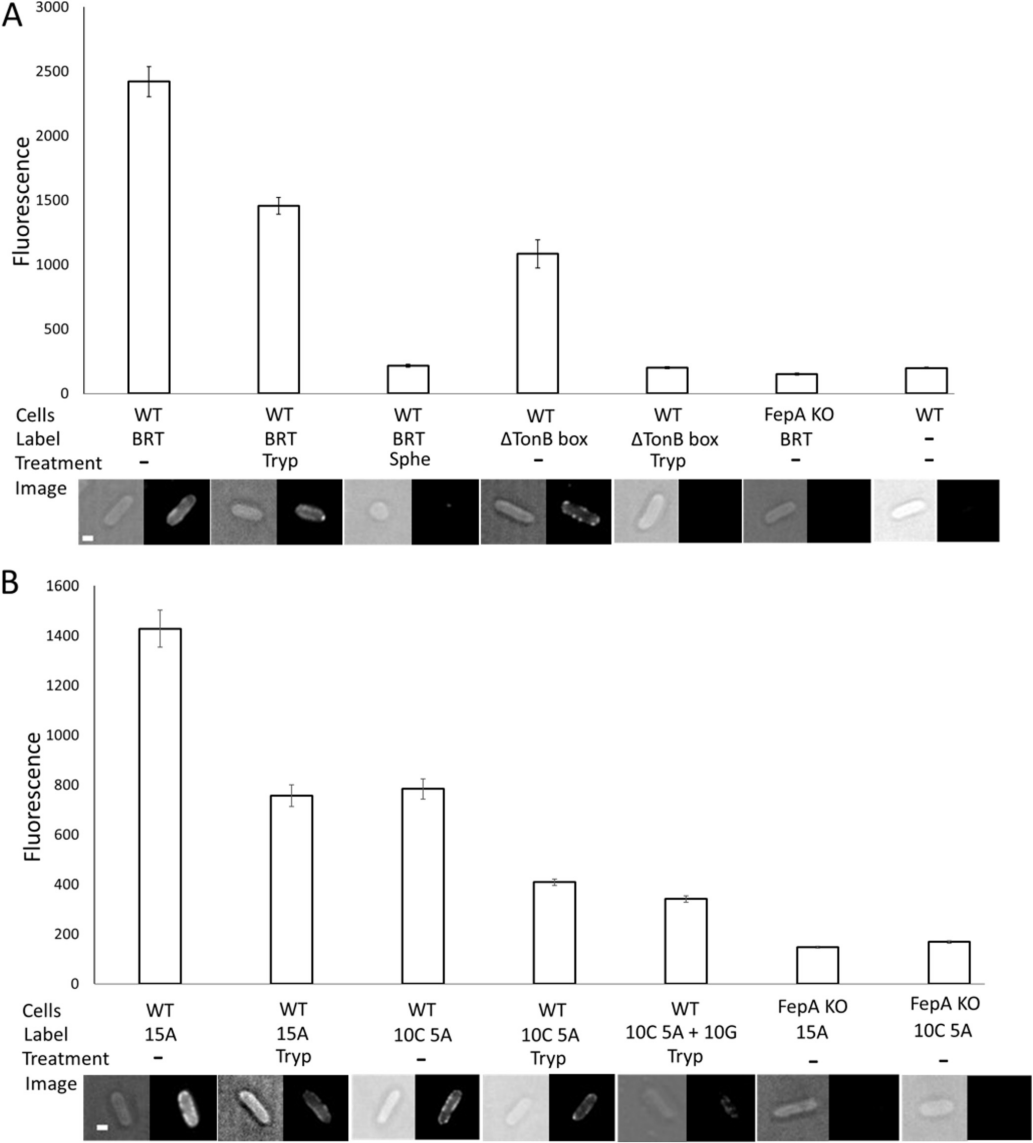
ssDNA follows the ColB-RT translocation path into E. coli cells. (A) Translocation of ColB-RT-Alexa 488 (BRT) or ColB-RT ΔTonB box–Alexa 488 (ΔTonB box) constructs into E. coli MG1655 cells (WT) or E. coli BW25113 ΔFepA (FepA knockout [KO]) cells grown in minimal medium to mid-log growth phase (OD_600_, ∼0.35). OM translocation was defined as fluorescent signal resistant to trypsin treatment (Tryp). Cytoplasmic localization was defined as fluorescent signal remaining after spheroplasting the cells, which results in the removal of the OM and the periplasmic peptidoglycan layer (Sphe). The averaged fluorescence intensities were calculated from at least 120 cells (30 cells × 4 biological repeats), and standard error bars of each treatment are shown. Representative cellular images are below each treatment. Scale bar, 1 μm. (B) Translocation of ColB-RT-DNA-fused constructs were ColB-RT-A_15_ Alexa 488 (15A), ColB-RT A_5_C_10_ Alexa 488 (10C 5A), and ColB-RT A_5_C_10_ Alexa 488 + G_10_ (A_5_C_10_ + G_10_).

Previous work has demonstrated that Pseudomonas aeruginosa-specific pyocins are capable of transporting fluorophores into target cells (21, 22). Whether this is also possible for colicins and E. coli has yet to be determined. To address this question, the ColB-RT domain (residues 1 to 341) was labeled with Alexa Fluor 488 through modification of a cysteine residue at its C terminus and entry into E. coli investigated by fluorescence microscopy (Fig. 1A). Addition of trypsin was used to distinguish between ColB bound at the outer membrane and that which had translocated into the cells. Deletion of the ColB TonB box (residues 17 to 21) resulted in the complete loss of colicin’s ability to translocate, consistent with its Ton dependence; however, it maintained its ability to bind FepA (Fig. 1A). No binding was detected in a *fepA* deletion strain (Fig. 1A). Pore-forming colicins translocate to the periplasm to elicit cell death. Consistent with the periplasm being the final destination of ColB, the fluorescent signal of ColB-RT was lost upon spheroplasting cells (Fig. 1A). Having established that ColB-RT could transport a fluorophore to the periplasm, we then investigated whether the colicin could be used to deliver macromolecules into the cell, specifically ssDNA. The C-terminal cysteine of ColB-RT was modified with fluorescently labeled ssDNA (A_15_ or A_5_C_10_). DNase treatment of the ssDNA-labeled ColB-RT released the fluorophore (see Fig. S5 in the supplemental material); hence, DNase treatment could be used to distinguish between surface-bound ColB-RT and that which had translocated to the periplasm. As with fluorescently labeled ColB-RT, ColB-RT conjugated to fluorescently labeled DNA translocated to the periplasm in an FepA-dependent manner (Fig. 1B). Addition of a G_10_ oligonucleotide to ColB-RT conjugated to A_5_C_10_ decreased the fluorescence signal detected in the periplasm (Fig. 1B), suggesting either that dsDNA is transported poorly or that dissociation of the second DNA strand slows the translocation process.

10.1128/mBio.01787-21.5 FIG S5 ColB-RT conjugation to a fluorescent 15 b ssDNA construct. ColB-RT has been attached directly to Alexa 488 (left) or via a 15-adenine single-strain DNA construct (center) or via a 5-adenine 10-cytosine ssDNA (right). Upper images, Alexa 488 fluorescence; lower, Coomassie blue protein stain. BRT, ColB-RT–Alexa 488; BRT-15 b, ColB-RT conjugated to 15 b ssDNA; BRT dimer, nonlabeled ColB-RT dimers mediated through the introduced C-terminal Cys. Download FIG S5, TIF file, 0.8 MB. Copyright © 2021 Cohen-Khait et al. 2021 Cohen-Khait et al. https://creativecommons.org/licenses/by/4.0/ This content is distributed under the terms of the Creative Commons Attribution 4.0 International license.

### Receptor FepA binding induces large-scale conformational changes in ColB.

**FIG 2:**
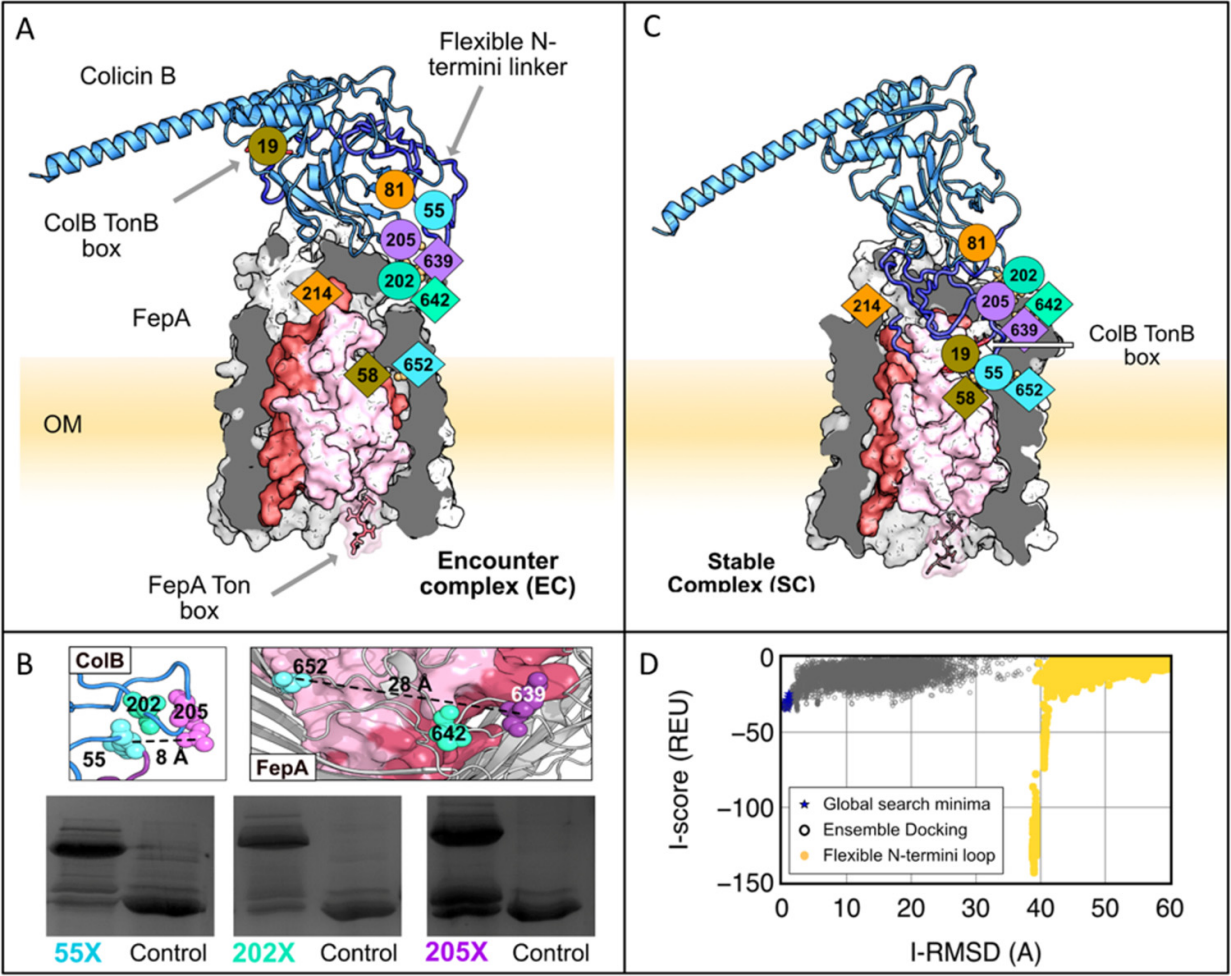
Structural insights on the ColB-FepA complex by pBPA cross-linking and Rosetta-based structural modeling. (A) Initial encounter complex (EC) modeled with moderate to little backbone flexibility (under 5 Å root mean square deviations [RMSD]). ColB (blue) and FepA (gray) form this encounter complex with *in vitro* cross-links, FepA-K639 and ColB-D202 (teal), and FepA-P642 and ColB-R205 (purple), which lie in proximity in the model. The last *in vitro* cross-link pair, FepA-S652 and ColB-Q55 (cyan), and the two *in vivo* cross-links, FepA-T58 and ColB-M19 (olive) and FepA A214 and ColB-G81 (orange), are not satisfied in this structure. (B) Mapped *in vitro* cross-linking sites on the ColB and FepA PDB structures (1RH1 and 1FEP, respectively). Cropped relevant cross-link gels. Self-cross-linking control to the right of each lane (full *in vitro* cross-linking image is in Fig. S3A). (C) Fully assembled spontaneously formed stable complex (SC) modeled with the Rosetta FlopyTail algorithm (48) simulating the partially unstructured ColB 1–55 as a floppy tail. (D) Rosetta interface score (*y* axis) versus interface RMSD (*x* axis) for output structures identified by local docking (ReplicaDock2) of ColB to FepA. RMSD is measured relative to the lowest-scoring global docking structure. There is a deep minimum resulting from the arrangement of the flexible N-linker for the FloppyTail models. Measurements corresponding to panel A are in navy blue, measurements corresponding to panel C are in yellow.

Many colicins bind multiple OM proteins or even multiple copies of the same OM protein (9). To address the question of whether additional proteins or copies of FepA were required for ColB transport, ColB complexes were assembled on the surface of ColB-sensitive E. coli, detergent extracted, and purified by nickel affinity, followed by size exclusion chromatography. Native mass spectrometry of this isolated complex revealed the FepA-ColB complex to have a 1:1 stoichiometry (Fig. S1), consistent with ColB binding and translocating through a single copy of FepA. No structures exist for the ColB-FepA complex. Hence, to understand how colicin associates with this TBDT, we resorted to a Rosetta docking algorithm (23), exploiting available PDB structures of unbound ColB (24) (PDB entry 1RH1) and FepA (18) (PDB entry 1FEP). The structures were initially positioned with the FepA extracellular loops facing the predicted ColB receptor-binding loops (25) (Fig. S2). This calculation revealed a clear energy funnel (Fig. 2D) for an encounter complex (EC) structure (Fig. 2A). As an independent test of the Rosetta model predictions we used pBPA cross-linking. We introduced p-benzoyl-l-phenylalanine (pBPA) mutations into ColB-RT surface loops, previously highlighted as potential FepA binding sites (25) (Fig. S2). Exposure to UV (365 nm) results in pBPA nonspecific cross-linking into C-H bonds within ∼4 Å (26). Photoactivated cross-linking experiments were performed both *in vitro*, using an OM protein fraction as a FepA source, and *in vivo*, using live E. coli cells. We identified cross-links by SDS-PAGE and further analyzed these by liquid chromatography-tandem mass spectrometry (LC-MS/MS), as previously described by White et al. (21). We identified three cross-links *in vitro*, two of which (ColB residues D202X and R205X with FepA residues P642 and K639, respectively) validated the EC computed by Rosetta (Fig. 2A and Fig. S3A and S4A to C), the success of the computational prediction is a likely consequence of recent progress in the Rosetta docking energy function (23, 27). However, a third cross-link, ColB residue Q55X with FepA residue S652, could not be explained by the computed EC. ColB Q55 is in close proximity (∼8 Å) to ColB D202 and R205 in the ColB PDB structure (PDB entry 1RH1), yet its mapped FepA cross-link appears 28 Å apart from the mapped cross-link of ColB 205 (Fig. 2B). This disagreement was suggestive of a conformational change accompanying formation of the complex. Hence, to improve the structural model of the ColB-FepA complex, we simulated the N-terminal portion of ColB (residues 1 to 55) as a floppy tail, allowing it to sample its environment freely (23). The resulting model of the stable complex (SC) now explains all three *in vitro*-observed cross-links (Fig. 1C) and is more energetically favorable than the initially calculated EC (Fig. 2D and Movie S1). The calculated SC also brings the ColB TonB box (residues 17 to 21) closer to the FepA lumen (Fig. 2C). In conclusion, using a combination of photoactivated cross-linking and Rosetta-based docking simulations, we have uncovered that ColB associates with its receptor/translocator FepA through an initial encounter complex that then rearranges to the final stable complex, which prepares the toxin for import.

10.1128/mBio.01787-21.1 FIG S1 ColB-FepA stoichiometric complex ratio as observed by native-state mass spectrometry. A clear charge state distribution corresponding to unbound FepA and ColB is observed as well as a 1:1 noncovalent complex composed of one copy of each protein. Charge-reduced species of FepA is also present at higher *m*/*z* and indicative of a gas phase-induced dissociation. Also observed is a low-abundance charge state distribution that corresponds to the 1:1 FepA-ColB complex with a discrete mass increase of approximately 4,172 Da. This may correspond to the binding of a single lipopolysaccharide molecule often observed with membrane proteins from the OM, but no further experiments were conducted to further identify the adduct of this low-abundance species. Diss` FepA, FepA molecules that have dissociated from a FepA-ColB complex during the run. Download FIG S1, TIF file, 0.2 MB. Copyright © 2021 Cohen-Khait et al. 2021 Cohen-Khait et al. https://creativecommons.org/licenses/by/4.0/ This content is distributed under the terms of the Creative Commons Attribution 4.0 International license.

10.1128/mBio.01787-21.2 FIG S2 Structural alignment of ColB-RT PDB entry 1RH1 (blue) and ColE7 T domain PDB entry 2AXC (wheat) and positions of *pBPA* incorporation (orange sticks). The average deviation between the corresponding atoms of ColB-RT and ColE7 (RMSD) is 2.384 as calculated by PyMol. Both ColB-RT and ColE7 T share a similar pyosin_S fold, yet ColB-RT is the only one forming a complex with FepA. *pBPA* has been incorporated mainly in ColB exclusive surface loops to examine their role in FepA binding. Download FIG S2, TIF file, 0.5 MB. Copyright © 2021 Cohen-Khait et al. 2021 Cohen-Khait et al. https://creativecommons.org/licenses/by/4.0/ This content is distributed under the terms of the Creative Commons Attribution 4.0 International license.

10.1128/mBio.01787-21.3 FIG S3 ColB-RT *pBPA* GFP cross-links to FepA. (A) A 12% SDS-PAGE gel emphasizing ColB-RT GFP-FepA cross-linking experiments *in vitro*. Cross-links were identified as significant size-shifted bands as observed for residues 55, 202, and 205. A self-cross-linking control (with no FepA presence) was run to the right of each lane. (B) ColB-RT GFP-FepA cross-linking experiments performed *in vivo*. Cross-links of residues 49 and 51 produced peptides that did not allow their mapping by LC MS/MS. (C) *In vivo* cross-linking experiments using TonB knockout cells (GFP fluorescence image). Download FIG S3, PDF file, 2.6 MB. Copyright © 2021 Cohen-Khait et al. 2021 Cohen-Khait et al. https://creativecommons.org/licenses/by/4.0/ This content is distributed under the terms of the Creative Commons Attribution 4.0 International license.

10.1128/mBio.01787-21.9 MOVIE S1 SC model. Download Movie S1, MPG file, 1.3 MB. Copyright © 2021 Cohen-Khait et al. 2021 Cohen-Khait et al. https://creativecommons.org/licenses/by/4.0/ This content is distributed under the terms of the Creative Commons Attribution 4.0 International license.

### ColB exploits FepA for its active translocation into the cell.

**FIG 3:**
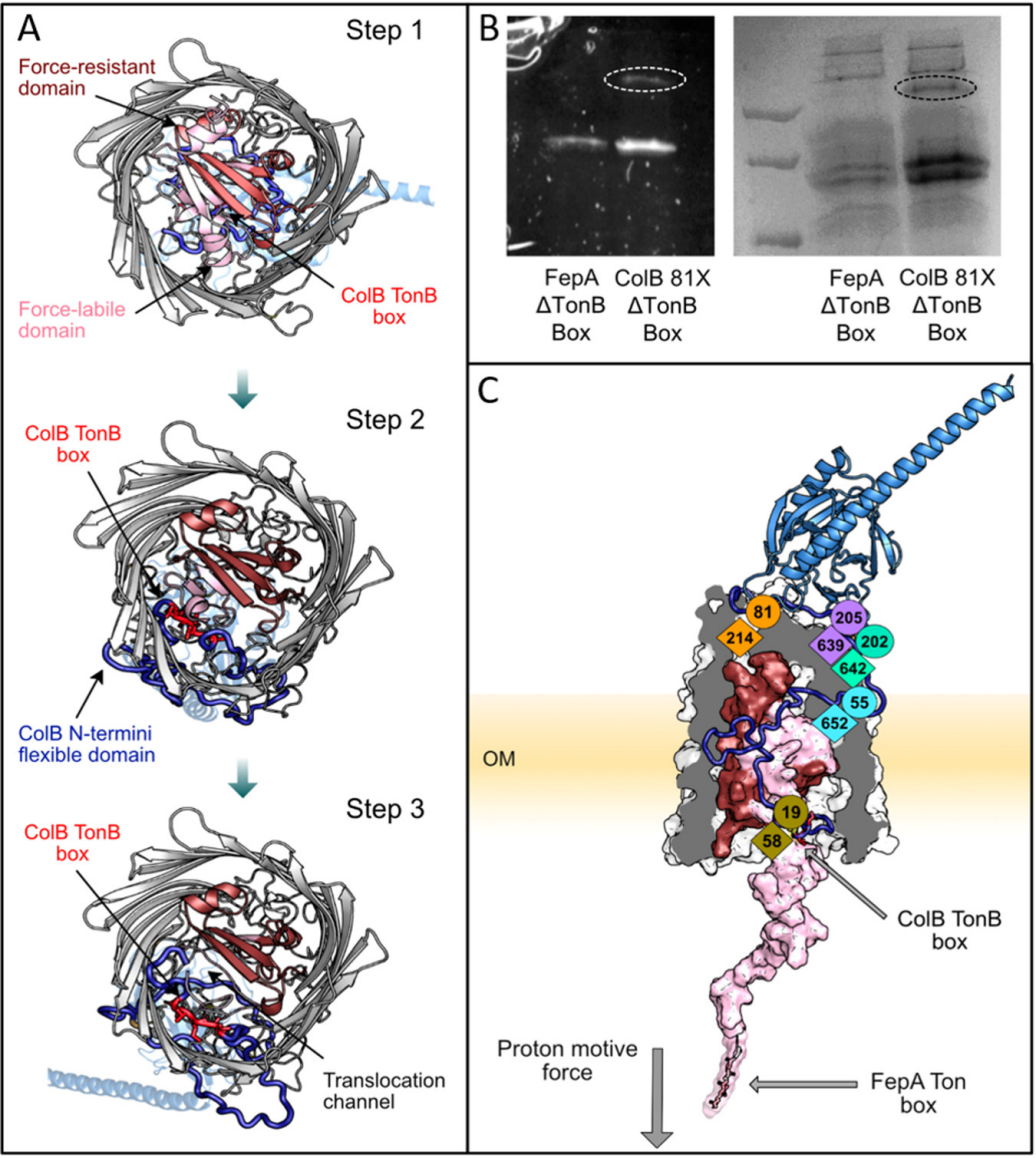
Partially unstructured ColB-RT 55-residue N-terminal end occupies the gap generated by the active unfolding of the FepA N-terminal half plug domain. (A) A bottom-to-top view of the hypothesized translocation pathway (stage 3) created with Rosetta by pulling the FepA N terminus into the cell. Step 1, SC complex is formed and the force-labile half-plug domain (light pink) begins to unfold. Step 2, the force-labile half-plug is partially unfolded, which allows the ColB N-terminal loop (blue) to occupy the void created by the absence of the plug domain. Step 3, the unfolding of the FepA half-plug domain creates a channel for the ColB N-terminal loop to enter. (B) The ability of ColB-81X GFP to cross-link *in vivo* as a function of both ColB and FepA TonB boxes. GFP fluorescence (right) and Coomassie blue stain (left) are shown. Cross-linked band is circled. (C) The top-scoring model portraying the translocation state (step 3). In this final-stage model, all the cross-link constraints are satisfied and the model is energetically favorable over other states (see Fig. S7 for energy calculations).

The route taken by ColB during FepA-dependent translocation is unknown. Here, we show how the partially unstructured flexible N-terminal tail of ColB (residues 1 to 55) occupies the channel generated by the TonB-dependent unfolding of the N-terminal half of the FepA plug domain (Fig. 3). While complex formation (Fig. 2) is a highly specific step, the translocation mechanism through 22 stranded beta-barrel TBDTs is likely to be applicable to many other systems sharing similar protein folds (Fig. S6). The three *in vitro* cross-links we obtained were also observed *in vivo* as well as two additional cross-links (ColB M19X and G81X with FepA T58 and A214), which we further mapped by LC-MS/MS (Fig. S3B, Fig. S4D and E). The additional two cross-links did not form in the absence of the energy-transferring protein TonB (Fig. S3C).

10.1128/mBio.01787-21.4 FIG S4 LC MS-MS analysis of the ColB–FepA cross-links (from Fig. S3). (A) LC MS-MS spectra of ColB-RT 55X GFP *in vitro*-identified cross-link indicating association with FepA 652Y. (B) LC MS-MS spectra of ColB-RT 202X GFP *in vitro*-identified cross-link indicating association with FepA 639K. (C) LC MS-MS spectra of ColB-RT 205X GFP *in vitro*-identified cross-link indicating association with FepA 642P. (D) LC MS-MS spectra of ColB-RT 19X GFP *in vivo*-identified cross-link indicating association with FepA 58T. (E) LC MS-MS spectra of ColB-RT 81X GFP *in vivo*-identified cross-link indicating association with FepA 214A. Download FIG S4, PDF file, 2.5 MB. Copyright © 2021 Cohen-Khait et al. 2021 Cohen-Khait et al. https://creativecommons.org/licenses/by/4.0/ This content is distributed under the terms of the Creative Commons Attribution 4.0 International license.

10.1128/mBio.01787-21.6 FIG S6 FepA and ColB-RT are representatives of common protein folds. (A) Structural alignment of FepA and 12 additional 22-stranded β-barrel OM bacterial proteins identified by the MADOKA server summarized in the presented table. Side view of the alignment (left), bottom view (right) of N-terminal half plug domain in pink, C-terminal half plug domain in hot pink. (B) Structural alignment of ColB (blue) with ColE7 (gray) and ColE3 (green) N-terminal translocation domains identified by Pfam as the pyocin_S domain superfamily. Table summarizing alignment details is on the right. Download FIG S6, PDF file, 0.4 MB. Copyright © 2021 Cohen-Khait et al. 2021 Cohen-Khait et al. https://creativecommons.org/licenses/by/4.0/ This content is distributed under the terms of the Creative Commons Attribution 4.0 International license.

10.1128/mBio.01787-21.7 FIG S7 Interface score v/s l-RMSD (Å) for all the decoys generated for the docking simulations. The global docking minima (blue) are obtained from global docking runs for reference. Stage 1 ensemble docking models (to create EC) are represented in gray, and the stage 2 models (for SC) obtained with FloppyTail are represented in yellow. Stage 3 models involving 3 steps of half-plug unfolding are represented in green (step 1), teal (step 2), and purple (step 3), respectively. As the half-plug is completely unfolded, the interface energies of colicin B in a partial translocation stage with FepA has a deeper energy well than the stage 2 encounter complex. Download FIG S7, TIF file, 0.09 MB. Copyright © 2021 Cohen-Khait et al. 2021 Cohen-Khait et al. https://creativecommons.org/licenses/by/4.0/ This content is distributed under the terms of the Creative Commons Attribution 4.0 International license.

The TonB box of TBDTs and bacteriocins is a conserved pentapeptide sequence essential for interaction with TonB (28). Two TonB boxes participate in the ColB translocation process: one on colicin itself and the other on its OM receptor, FepA (16, 29). We examined the ability of the *in vivo* observed ColB 81-FepA 214 cross-link to form as a function of both the FepA and ColB TonB boxes. The ColB 81-FepA 214 cross-link did not form in the absence of the FepA TonB box, but it still formed in the absence of the ColB TonB box (Fig. 3B). Hence, as both TonB boxes are essential for full colicin translocation, the ColB 81-FepA 214 cross-link appears to capture a stable intermediate translocation step. These experiments were not performed on the second *in vivo*-identified cross-link ColB 19-FepA 58, as ColB 19 is already part of the ColB TonB box.

To investigate the structures during the dynamic translocation process, we applied Rosetta to simulate the unfolding of the N-terminal half (residues 1 to 74) of the globular FepA plug domain, as previously demonstrated for BtuB (30). We simulated the ColB-FepA translocation process starting with the computed SC structure (Fig. 2C) and using the *in vivo*-identified cross-links as guides to generate three intermediate structures in 4-Å increments (Fig. 3A and C). The simulated structures suggest that the translocating N-terminal ColB tail (residues 1 to 55) occupies the cavity generated by the FepA half plug removal with the ColB TonB box now positioned in place of the former FepA TonB box (Fig. 3A and C).

## DISCUSSION

The OM of Gram-negative bacteria excludes several classes of antibiotics (31). As a means of subverting this impermeability, Trojan horse antibiotics rely on conjugating antibiotic moieties to siderophores that are actively imported into cells via TBDTs (32, 33). Here, we show, using fluorescence microscopy, that the FepA-specific bacteriocin ColB can similarly transport large cargo molecules into E. coli under the force of the PMF (Fig. 1). We also elucidate the mechanism by which ColB binds to FepA and uses the TBDT to translocate across the OM.

ColB was one of the earliest colicins to be identified (34), yet how this bacteriocin, and its close homologue ColD, recognize FepA has been unclear until now. Using photoactivated cross-linking combined with Rosetta-based simulations, we show that association involves at least two steps in which an initial encounter complex is formed that then rearranges. The conformational change involves the flexible N-terminal end of the colicin (residues 1 to 55) moving by up to 62 Å to form the final stable complex. An important consequence of these conformational changes is that they poise the ColB Ton box close to the channel that subsequently appears during PMF-mediated activation of the TBDT by TonB in the inner membrane. While previous studies have demonstrated that the Ton boxes of both ColB and FepA are important for import (16, 29), they do not report on the sequence of events where they are deployed. *In vivo* cross-linking data reveal that the cross-link between ColB-RT G81X and FepA A214 requires the FepA Ton box but not that of ColB (Fig. 3B and Fig. S3B and S4E), consistent with this cross-link reporting on activation of the TBDT by the PMF. The involvement of the ColB Ton box must be subsequent to this, as has been shown for the import of pyocin S2 through its TonB-dependent transporter FpvAI in Pseudomonas aeruginosa (21).

Past chemical modification data have presented a contradictory picture as to whether ColB translocates across the E. coli OM by direct transfer through FepA (16, 35, 36). Transport of ColB through FepA would require at least partial unplugging of its central pore. Unplugging of a TBDT to enable uptake of a ligand has been demonstrated by atomic force microscopy for the vitamin B_12_ transporter BtuB. The N-terminal globular plug domain of BtuB is composed of two mechanically independent half-plug domains. The N-terminal half, which lies proximal to the Ton box, is more amenable to forced unfolding than the C-terminal half (30). We therefore simulated the unfolding of the N-terminal half-plug of FepA by analogy with that of BtuB (30). The computed model (Fig. 2C) emphasizes the importance of the two independent encounters with the energy-transferring protein TonB. The first receptor-mediated encounter allows the translocation of the ColB TonB box to the periplasm (Fig. 3C), while the second activates colicin translocation into the cell. The computed model also suggests that the 55-residue N-terminal end of the translocating colicin mimics the unfolded receptor half-plug and, indeed, replaces the receptor’s TonB box with that from colicin (Fig. 3A and C).

In summary, the OM translocation of ColB is a highly dynamic process involving two association steps followed by two TonB-dependent events. Our simulations also suggest that colicin mimics the part of the FepA half-plug that is removed during import, thereby presenting its own Ton box to the periplasm. The translocation mechanism likely also applies to ColD, which binds FepA through a similar receptor-binding domain and is Ton dependent (37). The ability of bacteriocin-DNA conjugates to piggy-back the colicin into the cell opens a range of possibilities to utilize bacteriocins for bypassing the Gram-negative bacterial OM. This includes development of novel antibiotic delivery strategies and even genomic manipulations.

## MATERIALS AND METHODS

### Protein expression and purification.

All colicin constructs were conjugated to a 6×His tail at their C terminus and cloned at the second multiple cloning site of the pACYCDuet-1 (Novagen) plasmid, where they were expressed under a T7 promoter. The plasmids were transformed into BL21(DE3) E. coli cells. Transformed cells were grown at 37°C in lysogeny broth (LB), pH 7.2, while shaking at 180 rpm to an optical density at 600 nm (OD_600_) of ∼0.6, at which point 1 mM isopropyl-d-thiogalactopyranoside (IPTG) was added and the temperature was reduced to 20°C for an overnight incubation. Protein-expressing cells were resuspended in 20 mM Tris, pH 7.5, 0.5 M NaCl, 5 mM imidazole and sonicated (70%, 1.5 min, 3 s on, 7 s off; Sonicator 4000). The sonicated cell extract was spun down and the supernatant was incubated with His-binding resin (69670-5; Merck) for 10 to 30 min at room temperature. The Ni-resin and the bound protein were then gently (1,000 × *g*) spun down, washed three times, and resuspended in the same buffer containing 0.5 M imidazole that allowed protein elution. The protein was dialyzed to phosphate-buffered saline (PBS) at 4°C overnight. FepA was expressed on a pBAD/Myc-HisB (Novagen) plasmid transformed into either Bl21(DE3) or BW25113 ΔFepA(JW5086-3) E. coli cells. FepA has been expressed similarly to the colicin proteins, except for the LB growing medium pH being 6.12 and protein expression induction with 0.15% (wt/vol) l-arabinose. The FepA-containing OM fraction was purified as previously described for OmpF (38). Protein concentrations were determined through absorbance at 280 nm using a sequence-based extinction coefficient.

### Fluorescent labeling of ColB-RT.

Colicins were conjugated to Alexa Fluor 488 or 15 b DNA–Alexa Fluor 488 by maleimide reactions as described in Kleanthous et al. (39), with some adaptions: the purified protein was incubated with 10 mM dithiothreitol (DTT) for 1 h at room temperature (or overnight at 4°C) and was then run through a desalting column (5 ml HiTrap; buffer of 25 mM Tris, pH 7.5, 100 mM NaCl) and immediately incubated with a 1.1× or 3× ratio of maleimide DNA Alexa Fluor 488 conjugates (generated by Eurogentech) or maleimide Alexa 488, respectively, for 1 h at room temperature. The reaction was terminated by the addition of 5 mM DTT. The protein was desalted again, retrieved by Ni-beads as in the previous section, and dialyzed to PBS. The efficiency of the fluorescent conjugations was determined by absorbance measurements on a Jasco UV/VIS V-550. The protein-DNA conjugation sensitivity to DNase and trypsin treatments was analyzed on 15% SDS-PAGE gels.

### Microscopy.

The following E. coli strains were used for microscopy: MG1655 (wild type), BW25113 (wild type), and BW25113 (ΔFepA). A day prior to microscopy, single colonies of each strain were used to inoculate 10 ml LB and grown overnight at 37°C. Samples of 200 μl of each culture were transferred into 10 ml M9-glucose (2 mM MgSO_4_, 0.1 mM CaCl_2_, 0.4% [wt/vol] d-glucose) and grown at 37°C for 2.5 h until an OD_600_ of ∼0.35, at which point 34 μg/ml chloramphenicol was added to stop further cellular division. Forty-five minutes later the culture was aliquoted to 1-ml treatment tubes to which 100 nM fluorescent protein label was added and incubated with the culture for 1 h at 37°C. The cells were then washed three times in PBS. All different treatments (simple labeling/trypsin-treated cells/spheroplasted cells) were resuspended in 10 μl PBS (with 0.5 M sucrose and 20 mM MgCl_2_ for spheroplasted cells), of which 5 μl was placed onto 1% agarose pads (containing 0.5 M sucrose and 20 mM MgCl_2_ for the spheroplasts). All imaging was conducted on the ONI Nanoimager S. A 473-nm laser was used at 20% laser power to visualize cells labeled with Alexa 488. For each field of view, 10 frames were collected at an exposure of 100 ms. For data analysis purposes for each field of view, the 10 frames collected were averaged, and the fluorescent intensity of the cells and of their surrounding background was measured. Data analysis was performed by ImageJ software. Thirty cells and adjusted background intensities were analyzed for each treatment at each experiment. Each experiment was repeated 2 to 4 times on newly grown labeled and treated cells.

### Trypsin and spheroplast treatment for microscopy.

Trypsin treatment was applied on cells that had undergone the labeling procedure described in the previous section to determine whether the fluorescent signal translocated into the cells. The cells were incubated with 1 mg/ml trypsin in PBS, pH 7.8, for 1 h at 37°C. The trypsin was then washed two times in PBS. To determine whether the fluorescent signal translocated into the cytoplasm, the cells were further spheroplasted. The trypsin-treated cells were resuspended in 0.8 M Tris, pH 8, 0.5 M sucrose, 1 mg/ml lysozyme, 2 mM EDTA for 30 min at room temperature, and 0.1 mg/ml trypsin was then added and incubated with the mixture for an additional 30 min. The cells where then washed in PBS, 0.5 M sucrose, 20 mM MgCl_2_.

### Native-state electrospray ionization mass spectrometry.

Sixty milligrams of ColB-RT (341 amino acids [aa]) were added to a 5-liter culture of BW25113(ΔFepA) cells overexpressing FepA from a pBAD/Myc-HisB (Novagen) plasmid. The complex was purified by following the protocol previously described for OmpF (38). A 5-ml HiTrap desalting column (GE Healthcare) was used to exchange the complex buffer into 100 mM ammonium acetate, 1% (wt/vol) *n*-octyl-β-d-glucopyranoside (β-OG), pH 6.9. Mass spectrometry measurements were made from a static nanospray emitter using gold-coated capillaries prepared in-house (40) on a quadrupole time-of-flight mass spectrometer (Micromass) modified for high mass transmission. Liberation of the protein complex from β-OG detergent required energetic instrument parameters, and the low *m/z* region of spectra was dominated by detergent clusters. Operating conditions used include capillary voltage of 1,800 V, sample cone of 200 V, extractor of 10 V, collision cell energy of 140 to 200 V, source backing pressure of 5.92 × 10^−3^ mbar, and argon collision cell pressure of 3.5 to 5 MPa.

### Cross-linking.

The cross-linking procedure was similar to that of White et al. (21). In short, *pBPA* mutations were introduced at 21 different positions of ColB-RT (341 aa) green fluorescent protein (GFP). For *in vitro* cross-linking, 1 μM *pBPA* containing colicin was incubated with 1 ml of an OM protein fraction (in PBS, pH 6.5, 5 mM EDTA, 2% β-OG) extracted from BW25113 FepA knockout cells over expressing FepA containing ∼1 μM FepA and exposed to UV light (365 nm) for 1 h at 4°C. The colicin and bound/cross-linked FepA were then extracted by EDTA-resistant Ni-beads cOmplete (Merck). For *in vivo* cross-linking, the colicin was incubated with 800 ml cells that overexpressed FepA through an overnight incubation at 20°C. The *pBPA* containing colicin was added to the LB medium (pH 6.12) and incubated for 90 min at 37°C while shaking. The cells were then spun down (3,000 × *g*, 20 min, 4°C), colicin excess was washed with 50 ml PBS, the cells were resuspended in 10 ml PBS and exposed to UV light (365 nm) for 1 h at 4°C. The cells were then resuspended in 10 mM Tris, pH 8, 0.25% lithium diiodosalicylic acid (LIS), 2% Triton X-100, sonicated, the cell debris were spun down (10,000 × *g*, 10 min, 4°C), and the supernatant ultracentrifuged (200,000 × *g*, 45 min, 4°C). The pellet was resuspended in PBS, pH 6.5, 5 mM EDTA, 2% β-OG, ultracentrifuged again, and the colicin with its bound/cross-linked proteins was extracted by EDTA-resistant Ni-bead cOmplete (Merck). The extracted proteins were run on 12% SDS-PAGE gels, and GFP fluorescent bands of adequate size were analyzed by LC-MS/MS for cross-linking mapping.

### LC-MS/MS cross-linking analysis.

Peptides were separated on an EASY-nLC 1000 ultrahigh-performance liquid chromatography (UHPLC) system (Proxeon) and electrosprayed directly into a Q Exactive mass spectrometer (Thermo Fisher). Peptides were trapped on a C_18_ PepMap100 precolumn (300-μm inner diameter by 5 mm, 100-Å pore size; Thermo Fisher) using solvent A (0.1% [vol/vol] formic acid in water) at 500 × 10^5^ Pa and then separated on an in-house-packed analytical column (50 cm by 75-μm inner diameter packed with ReproSil-Pur 120 C_18_-AQ, 1.9 μm, 120 Å pore size; Maisch GmbH) with a linear gradient from 10% to 55% (vol/vol) solvent B (0.1% [vol/vol] formic acid in acetonitrile) in 45 min at 200 nl/min. Full-scan MS spectra were acquired in the Orbitrap (scan range, 350 to 2,000 *m/z*; resolution, 70,000; automatic gain control target, 3e6; maximum injection time, 100 ms). After the MS scans, the 10 most intense peaks were selected for higher-energy collisional dissociation (HCD) fragmentation at 30% of normalized collision energy. HCD spectra were also acquired in the Orbitrap (resolution, 17,500; automatic gain control target, 5e4; maximum injection time, 120 ms) with first fixed mass at 100 *m/z*. Charge states 1+ and 2+ were excluded from HCD fragmentation. MS data were searched using the pLink software (41). The database contained the target proteins and common contaminants. Search parameters were the following: maximum number of missed cleavages, 2; fixed modification, carbamidomethyl-Cys; variable modification 1, oxidation-Met; variable modification 2, Glu to pyro-Glu. Cross-linking from D to K, S, T, or N terminus was considered. Data were initially filtered to a false discovery rate (FDR) of 1%. Cross-links were further filtered/inspected with specific emphasis on fragmentation patterns.

### Structural modeling.

**(i) Computational modeling of the FepA-ColB interaction: structure preparation.** The crystal structures of ColB (1RH1 [24]) and FepA (1FEP [18]) were used as starting templates for the computational modeling. Because the crystal structures were missing key loops needed to effectively propagate backbone motions, we added these loops (residues 31 to 44 on ColB and 323 to 335 and 384 to 40 on FepA) using SWISS MODELLER (42). To eliminate energetically unfavorable side chain or backbone clashes, we then relaxed the structures using constraints to the native crystal coordinates using RosettaRelax (43).

**(ii) Stage 1: modeling the semirigid EC.** We determined putative local binding conformations by first performing rigid-body global docking using Rosetta’s ReplicaDock2 protocol (built upon prior work on temperature and Hamiltonian replica exchange Monte Carlo approaches [44, 45]) and clustering the lowest-energy docked structures. Starting from each low-energy structure, we refine the structures in a local binding region by using our RosettaDock4.0 (46) protocol that adaptively swaps receptor and ligand conformations from a pregenerated ensemble of structures. We diversify the backbone conformations in the ensemble by using (i) ReplicaDock 2.0, (ii) Rosetta Relax (43), and (iii) Rosetta Backrub (47). Local docking generates ∼6,000 decoys, which are scored based on their interface energies, defined as the energy difference between the total energy of the complex and the total energy of the monomers in isolation (see the supplemental material for details and command lines).

**(iii) Stage 2: modeling the SC, allowing backbone flexibility.** To explore the possibility of the ColB flexible N-terminal domain (residues 1 to 55) interacting explicitly with FepA, we used the Rosetta FloppyTail (48) algorithm, which allows modest sampling of backbone degrees of freedom following a two-stage approach. First, in the low-resolution stage, side chains are represented by a centroid atom and the backbone conformational space is extensively sampled. In the high-resolution stage, all side chain atoms then are returned to refine the structures. We generated ∼5,000 hypothetical decoys starting from the encounter complex obtained in stage 1 (EC). The 5,000 perturbation cycles and 1,000 refinement cycles were used for each decoy. To direct the MC sampling of the FloppyTail algorithm toward possible interacting regions, atom-pair constraints based on the experimental (*in vitro*) cross-linking residues guided the search. These constraints were calculated based on a harmonic potential with a mean of 6 Å and a standard deviation set to 0.25 Å between the Cα atoms of the candidate residues. Each output decoy was further relaxed to remove unfavorable clashes, and the 100 top-scoring models were then docked using RosettaDock4.0 (46) using a fixed backbone. Translational and rotational moves were performed on the top models to generate ∼5,000 docking decoys. To confirm the feasibility of these decoys, we evaluated the interface energies and compared the energy landscape of decoys in stage 2 with the prior decoys obtained in stage 1 (Fig. 1C).

**(iv) Stage 3: prediction of the translocation pathway applying *in vivo* cross-linking data.** Following the partial unfolding of the plug domain in the related TonB-BtuB system (30), we allowed backbone movement in the FepA 75-residue half-plug domain (residues 1 to 75) and the ColB flexible N-terminal domain (residues 1 to 43). Since simulating the dynamic unfolding of FepA half-plug with simultaneous translocation of the ColB via the barrel protein would be intensely demanding computationally, we instead create models to represent three steps along the dynamic pathway of the unfolding translocation process. A figure showing the workflow with intermediate snapshots and complete details of each phase of our three-part model creation are given in the supplemental computational methods. Briefly, to create each structure along the pathway, we (i) displace the FepA half-plug (residues 1 to 75) using Rosetta FloppyTail to pull the terminus out by 4, 8, and 12 Å, respectively, to begin making each of the three structural steps in the pathway; (ii) translocate the ColB N-terminal domain (residues 1 to 43) using both *in vitro* and *in vivo* cross-linking constraints with Rosetta FloppyTail; and (iii) refine both FepA and ColB conformation and rigid-body displacement using RosettaDock with a flexible FepA half-plug and ColB N-terminal domain. During stages 1 and 2, backbone motions in FloppyTail are propagated toward the closest terminus, but in stage 3, ColB backbone perturbations during docking are propagated back toward the bulk of ColB to facilitate it finding the optimal rigid-body displacement while the N-terminal domain is translocating. Finally, we calculate interface scores to reveal the favorability relative to conformations of other models presented in this paper along the hypothesized unfolding-translocation pathway (Fig. S7).

10.1128/mBio.01787-21.8 TEXT S1 Supplementary computational methods. Download Text S1, DOCX file, 0.6 MB. Copyright © 2021 Cohen-Khait et al. 2021 Cohen-Khait et al. https://creativecommons.org/licenses/by/4.0/ This content is distributed under the terms of the Creative Commons Attribution 4.0 International license.
