# Supplementary figures and images for: Colicin-Mediated Transport of DNA through the Iron Transporter FepA
Source: mBio. 2021 Sep 21;12(5):e01787-21. doi: 10.1128/mBio.01787-21 (PMC8546555; doi:10.1128/mBio.01787-21)

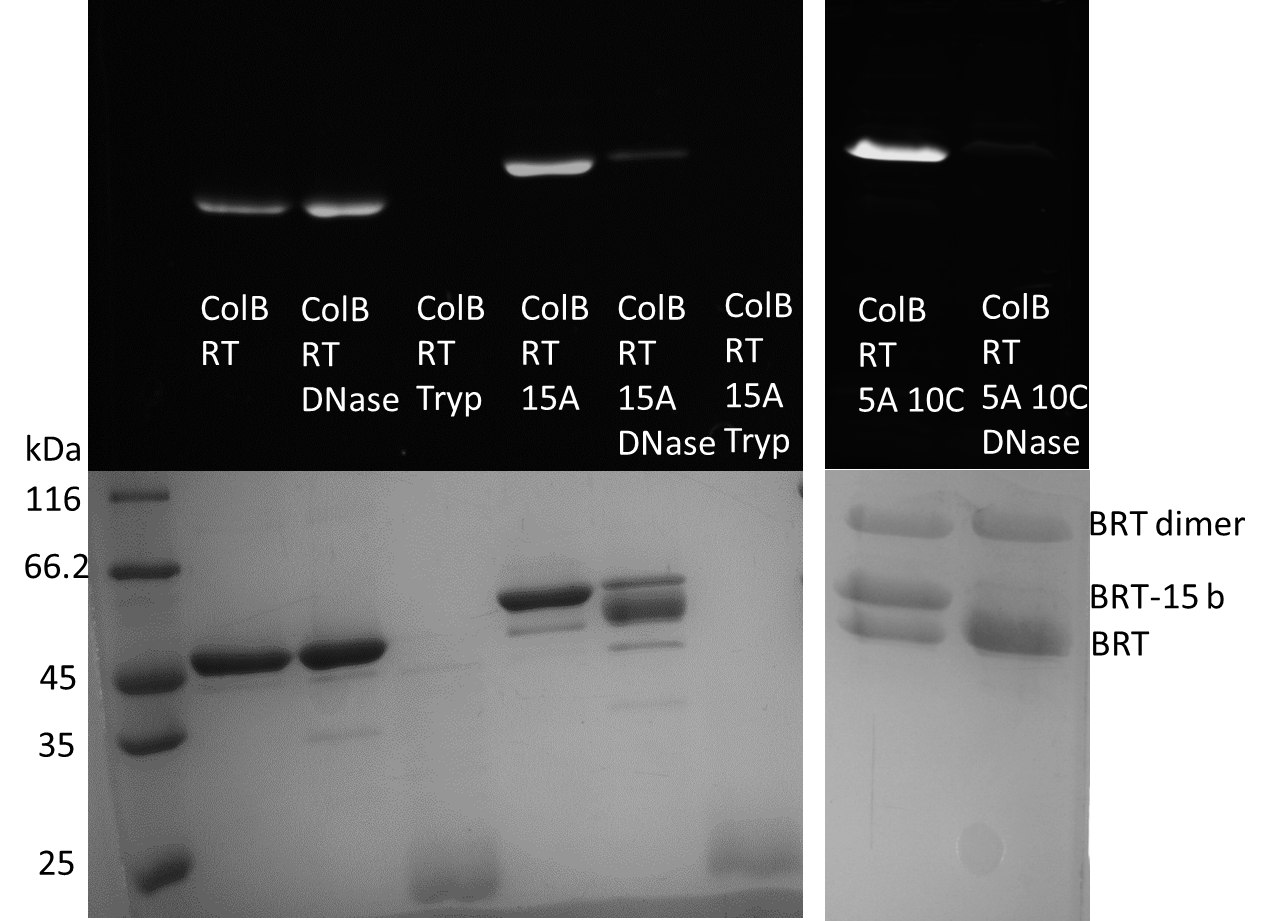

Supplement: FIG S5 [file mbio.01787-21-sf005.tif]

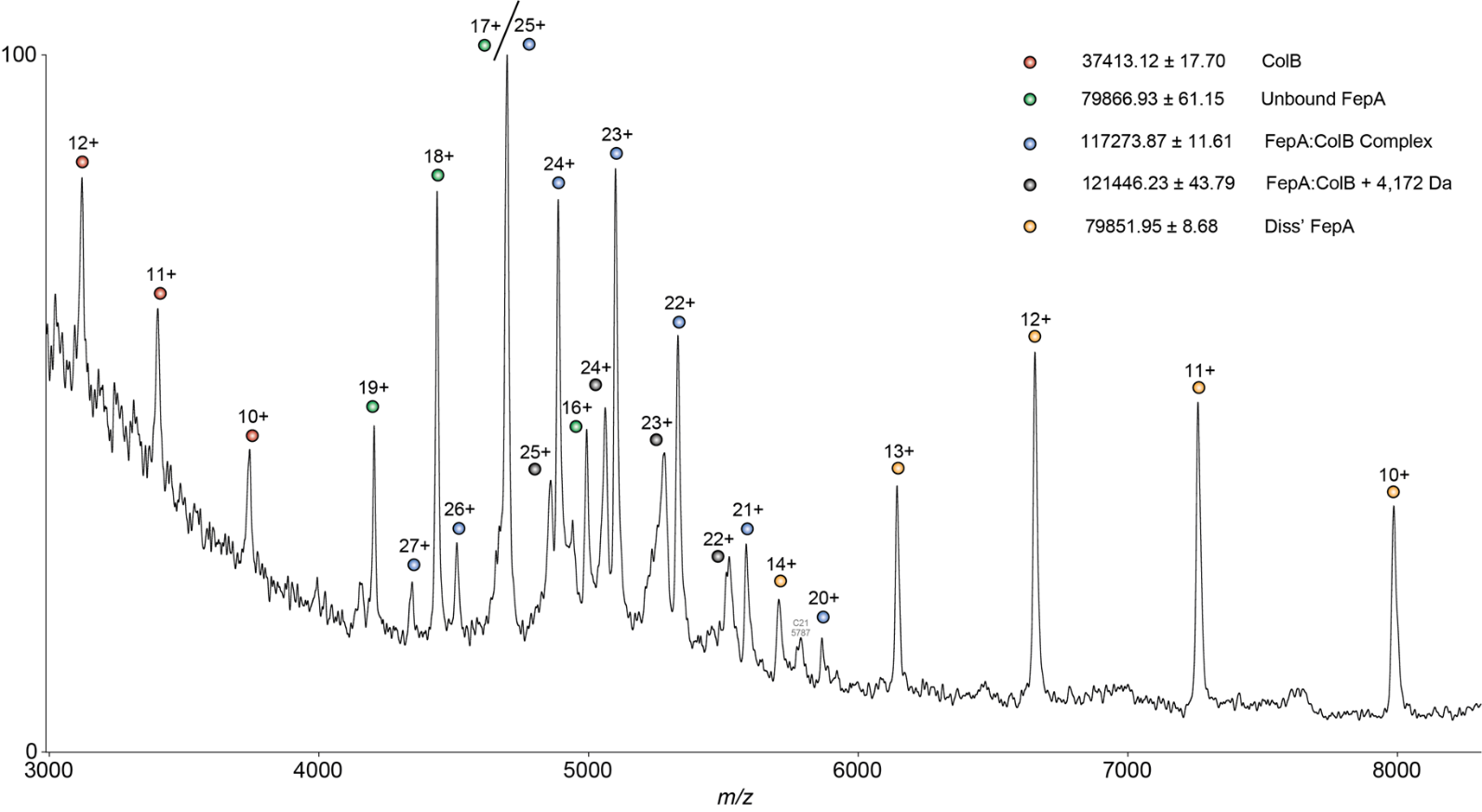

Supplement: FIG S1 [file mbio.01787-21-sf001.tif]

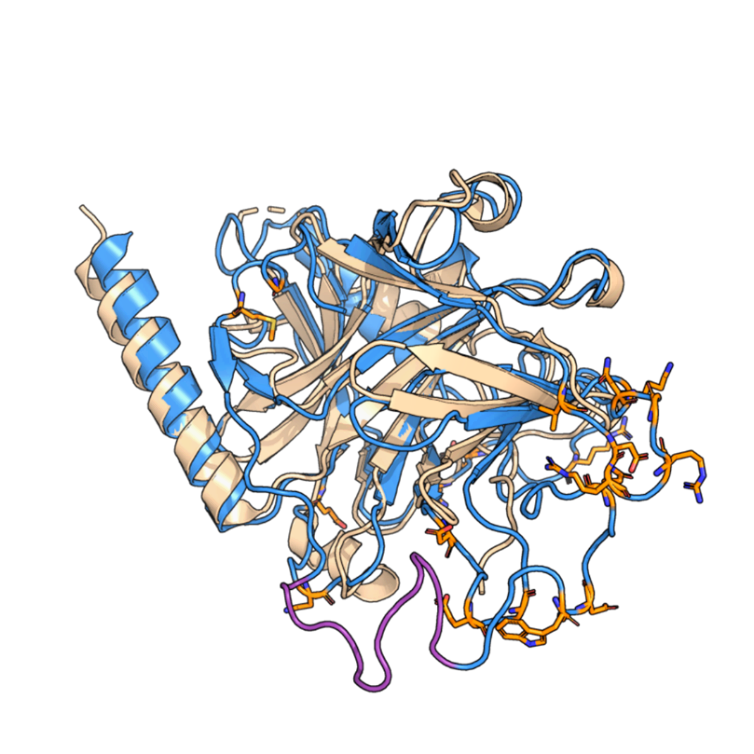

Supplement: FIG S2 [file mbio.01787-21-sf002.tif]

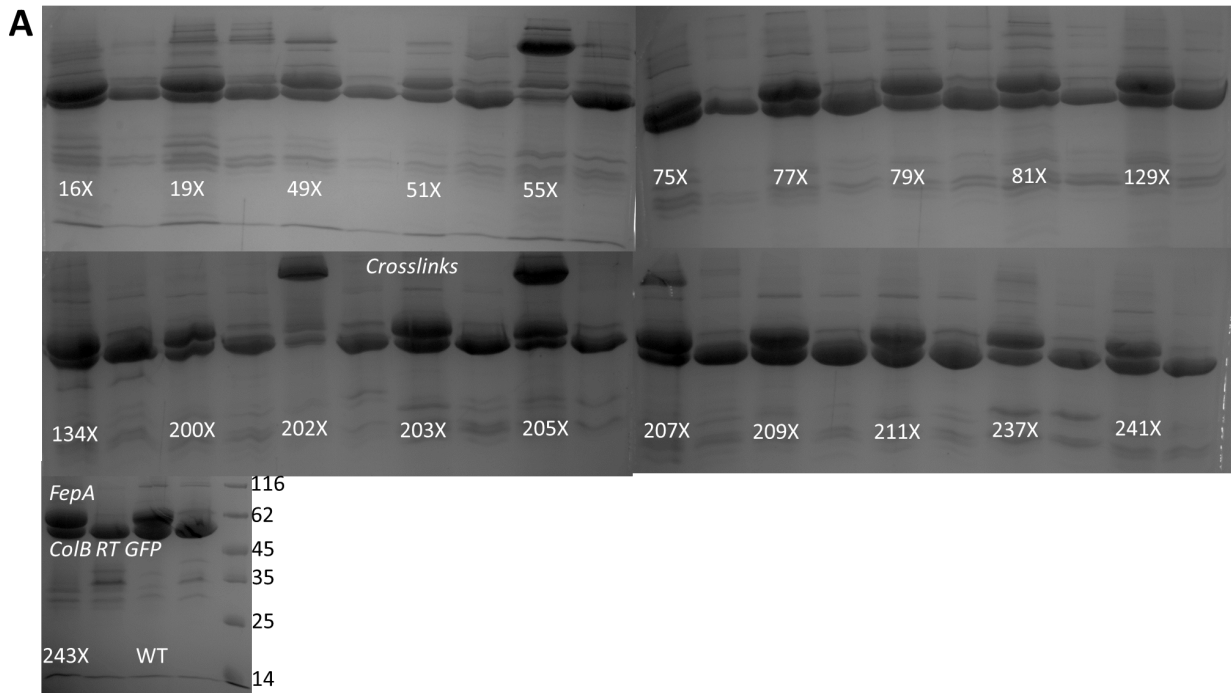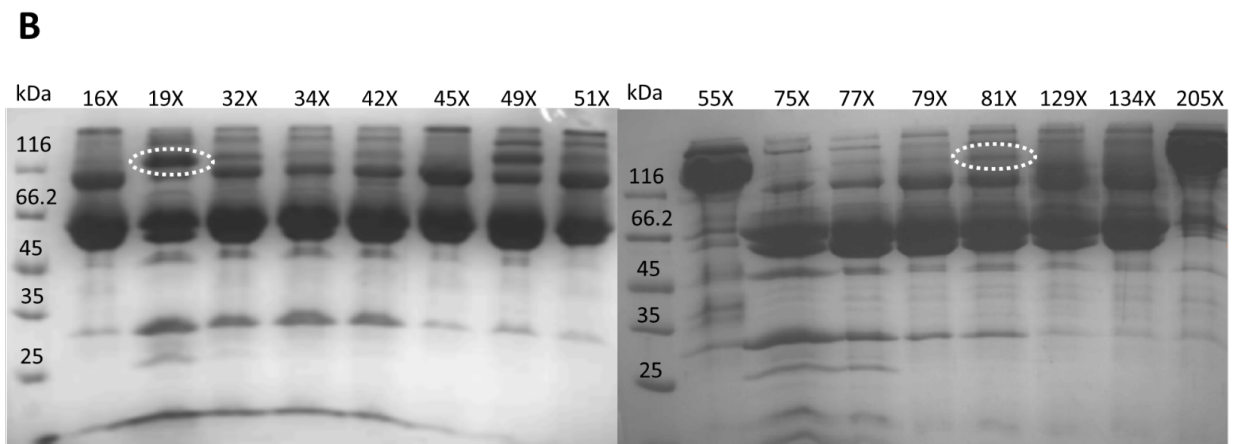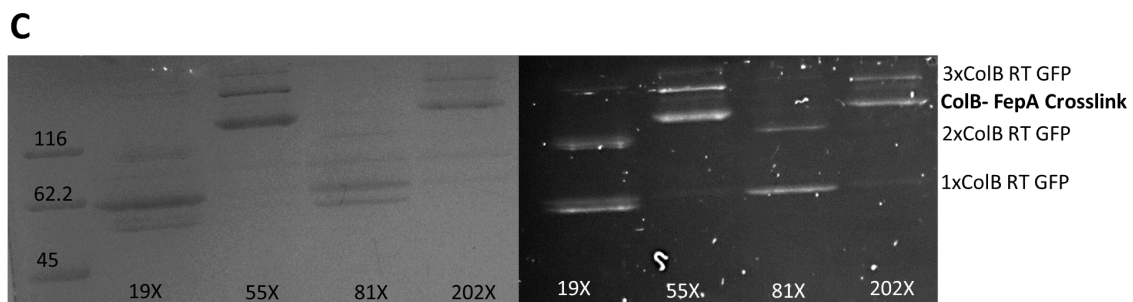

Supplement: FIG S6 [file mbio.01787-21-sf006.pdf]

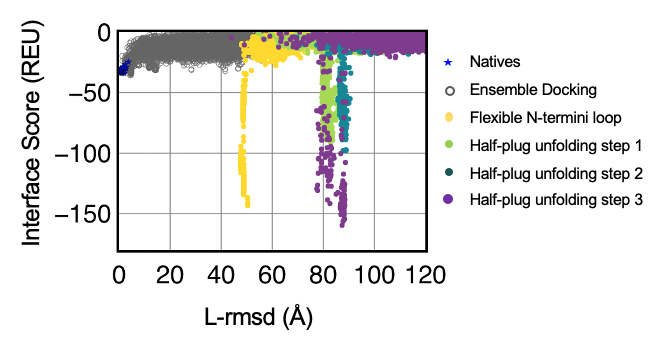

Supplement: FIG S7 [file mbio.01787-21-sf007.tif]
