# Supplementary material for: Colicin-Mediated Transport of DNA through the Iron Transporter FepA
Source: mBio. 2021 Sep 21;12(5):e01787-21. doi: 10.1128/mBio.01787-21 (PMC8546555; doi:10.1128/mBio.01787-21)
Supplement: FIG S3 [file mbio.01787-21-sf003.pdf]

A

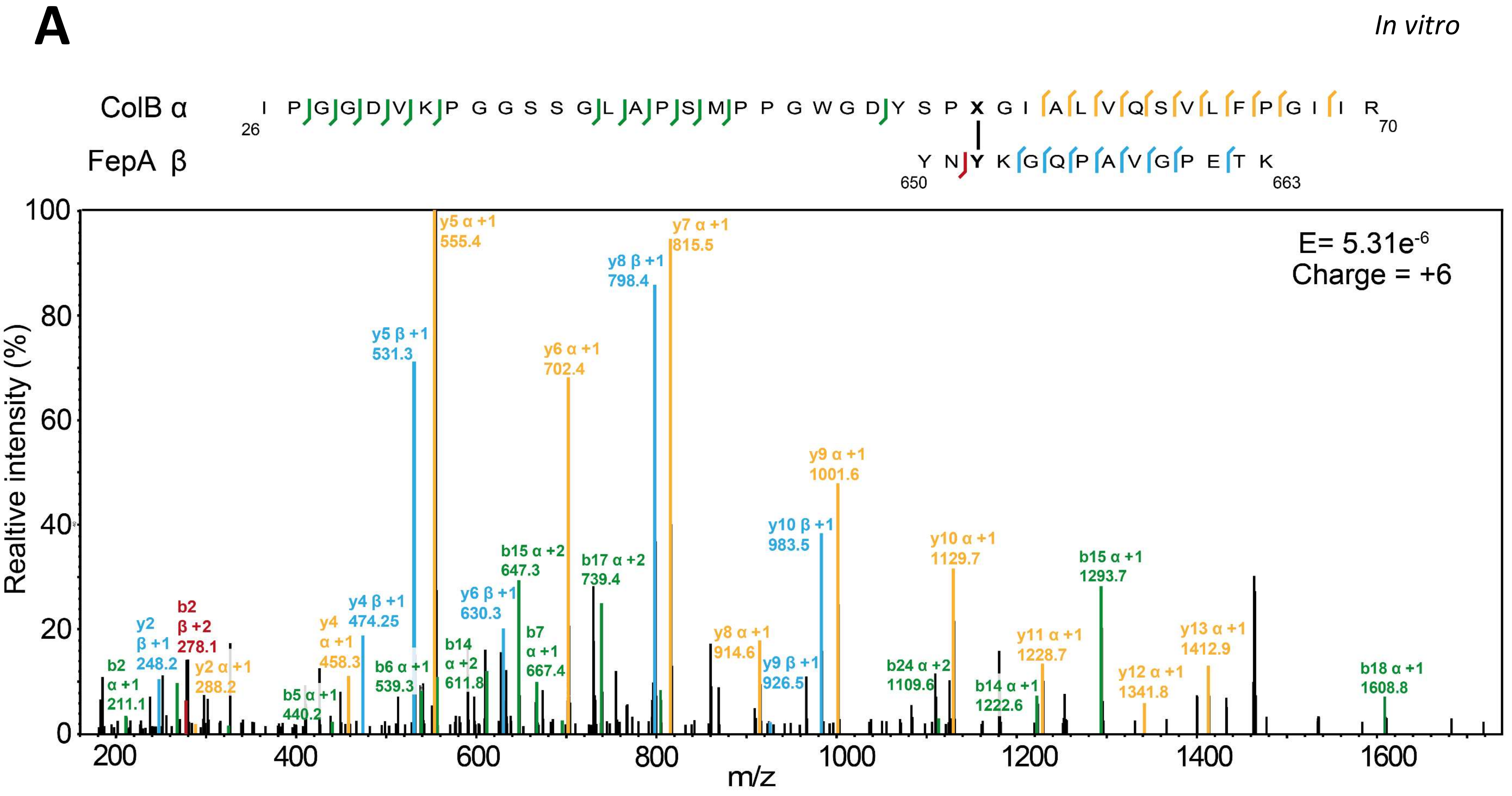

In vitro
ColB α
I P G G D V K P G G S S G L A P S M P P G W G D Y S P X G I A L V Q S V L F P G I I R
26
70
FepA β
Y N Y K G Q P A V G P E T K
650
663
E= 5.31e-6
Charge = +6
Realtive intensity (%)
m/z
y5 α +1 555.4
y7 α +1 815.5
y8 β +1 798.4
y5 β +1 531.3
y6 α +1 702.4
y9 α +1 1001.6
y10 β +1 983.5
y10 α +1 1129.7
b15 α +1 1293.7
b15 α +2 647.3
b17 α +2 739.4
y6 β +1 630.3
y4 β +1 474.25
b2 β +2 278.1
y2 β +1 248.2
b2 α +1 211.1
y2 α +1 288.2
y4 α +1 458.3
b5 α +1 440.2
b6 α +1 539.3
b14 α +2 611.8
b7 α +1 667.4
y8 α +1 914.6
y9 β +1 926.5
b24 α +2 1109.6
b14 α +1 1222.6
y11 α +1 1228.7
y12 α +1 1341.8
y13 α +1 1412.9
b18 α +1 1608.8

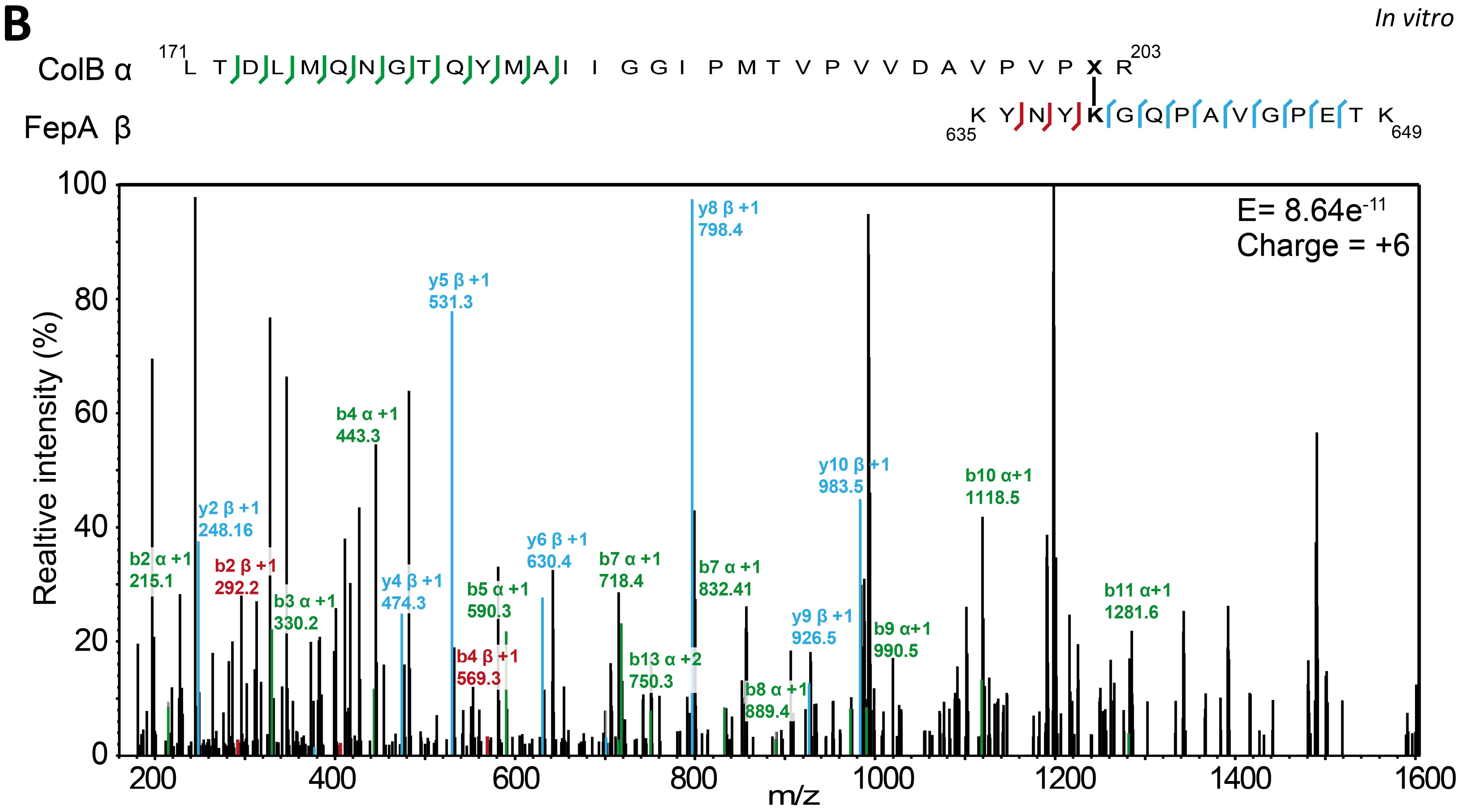
B
In vitro
ColB α
171 L T D L M Q N G T Q Y M A I I G G I P M T V P V V D A V P V P X R 203
FepA β
635 K Y N Y K G Q P A V G P E T K 649
E= 8.64e-11
Charge = +6
Realtive intensity (%)
m/z
y2 β +1 248.16
y4 β +1 474.3
y5 β +1 531.3
y6 β +1 630.4
y8 β +1 798.4
y9 β +1 926.5
y10 β +1 983.5
b2 α +1 215.1
b2 β +1 292.2
b3 α +1 330.2
b4 α +1 443.3
b4 β +1 569.3
b5 α +1 590.3
b7 α +1 718.4
b13 α +2 750.3
b7 α +1 832.41
b8 α +1 889.4
b9 α+1 990.5
b10 α+1 1118.5
b11 α+1 1281.6

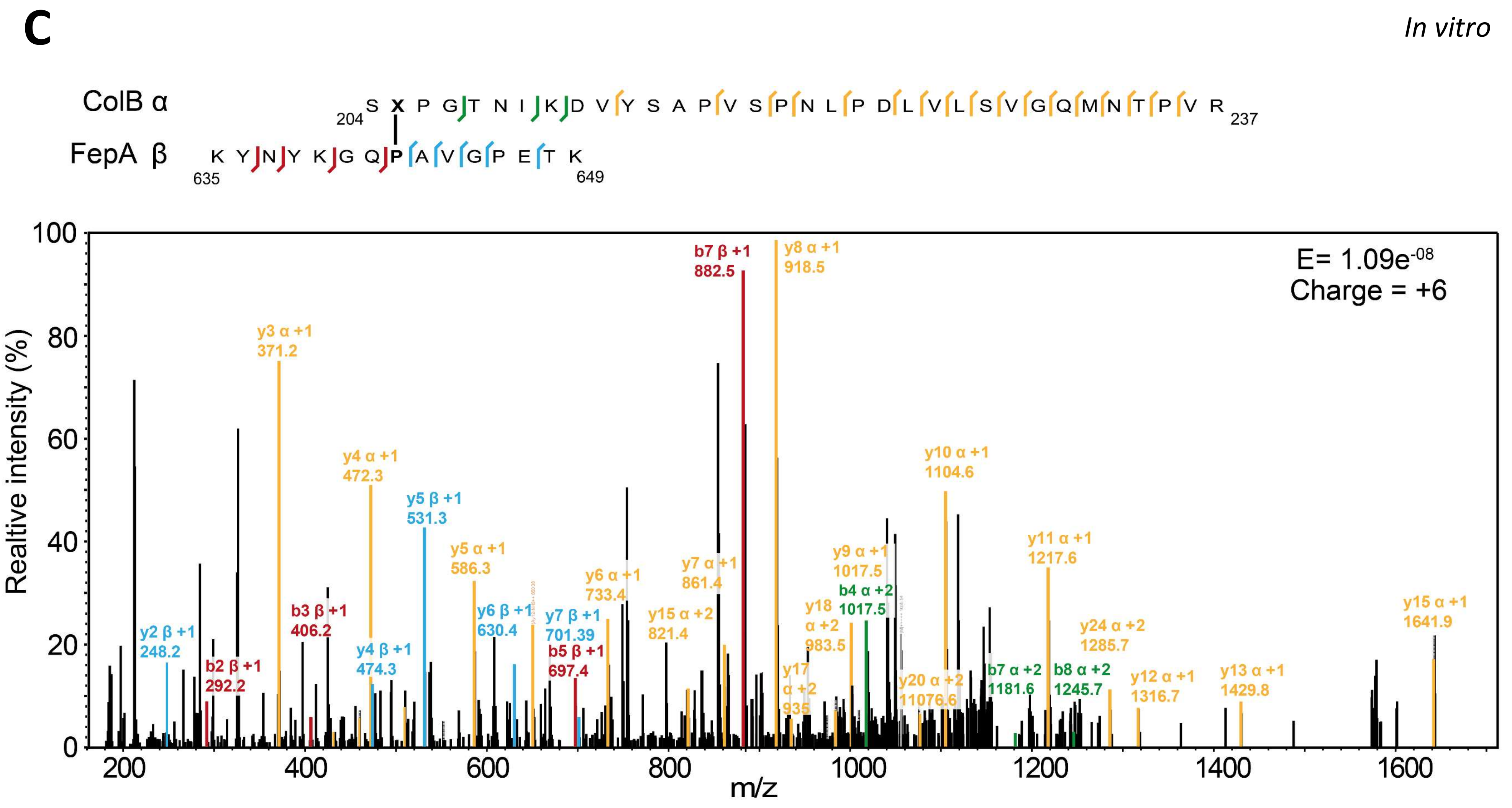
C
In vitro
ColB α
204 S X P G T N I K D V Y S A P V S P N L P D L V L S V G Q M N T P V R 237
FepA β
635 K Y N Y K G Q P A V G P E T K 649
E= 1.09e-08
Charge = +6
Realtive intensity (%)
m/z
b7 β +1 882.5
y8 α +1 918.5
y3 α +1 371.2
y4 α +1 472.3
y5 β +1 531.3
y5 α +1 586.3
y6 α +1 733.4
y7 α +1 861.4
y10 α +1 1104.6
y11 α +1 1217.6
y9 α +1 1017.5
b4 α +2 1017.5
y18 α +2 983.5
y15 α +1 1641.9
y24 α +2 1285.7
y2 β +1 248.2
b2 β +1 292.2
b3 β +1 406.2
y4 β +1 474.3
y6 β +1 630.4
y7 β +1 701.39
b5 β +1 697.4
y15 α +2 821.4
y17 α +2 935
y20 α +2 11076.6
b7 α +2 1181.6
b8 α +2 1245.7
y12 α +1 1316.7
y13 α +1 1429.8

D

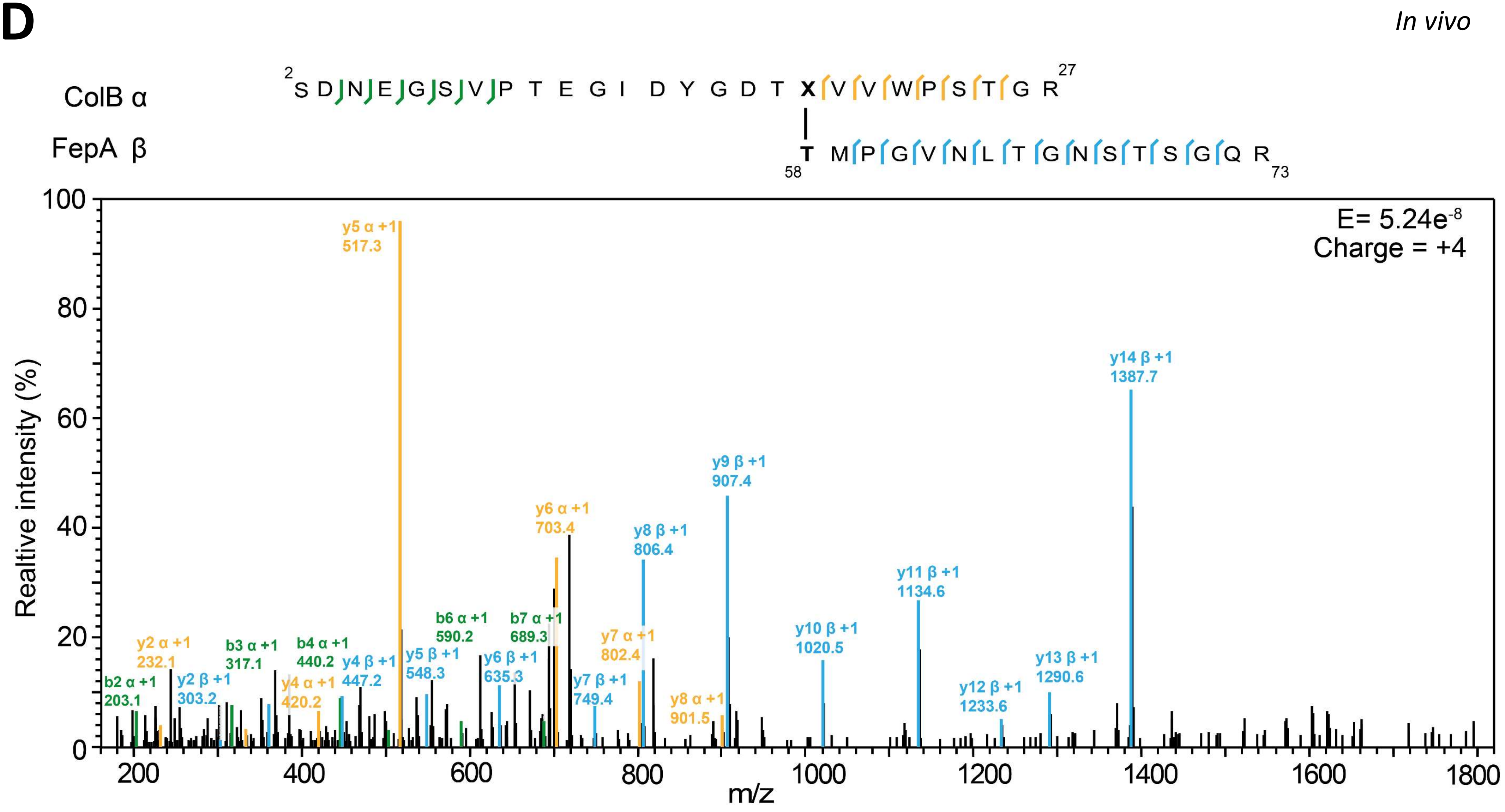

In vivo
ColB α
2 S D N E G S V P T E G I D Y G D T X V V W P S T G R 27
FepA β
58 T M P G V N L T G N S T S G Q R 73
E= 5.24e-8
Charge = +4
Realtive intensity (%)
m/z
y5 α +1 517.3
y14 β +1 1387.7
y9 β +1 907.4
y6 α +1 703.4
y8 β +1 806.4
y11 β +1 1134.6
y10 β +1 1020.5
y13 β +1 1290.6
y12 β +1 1233.6
y7 α +1 802.4
y8 α +1 901.5
y7 β +1 749.4
b6 α +1 590.2
b7 α +1 689.3
y6 β +1 635.3
y5 β +1 548.3
y4 β +1 447.2
b4 α +1 440.2
y4 α +1 420.2
b3 α +1 317.1
y2 β +1 303.2
y2 α +1 232.1
b2 α +1 203.1

E

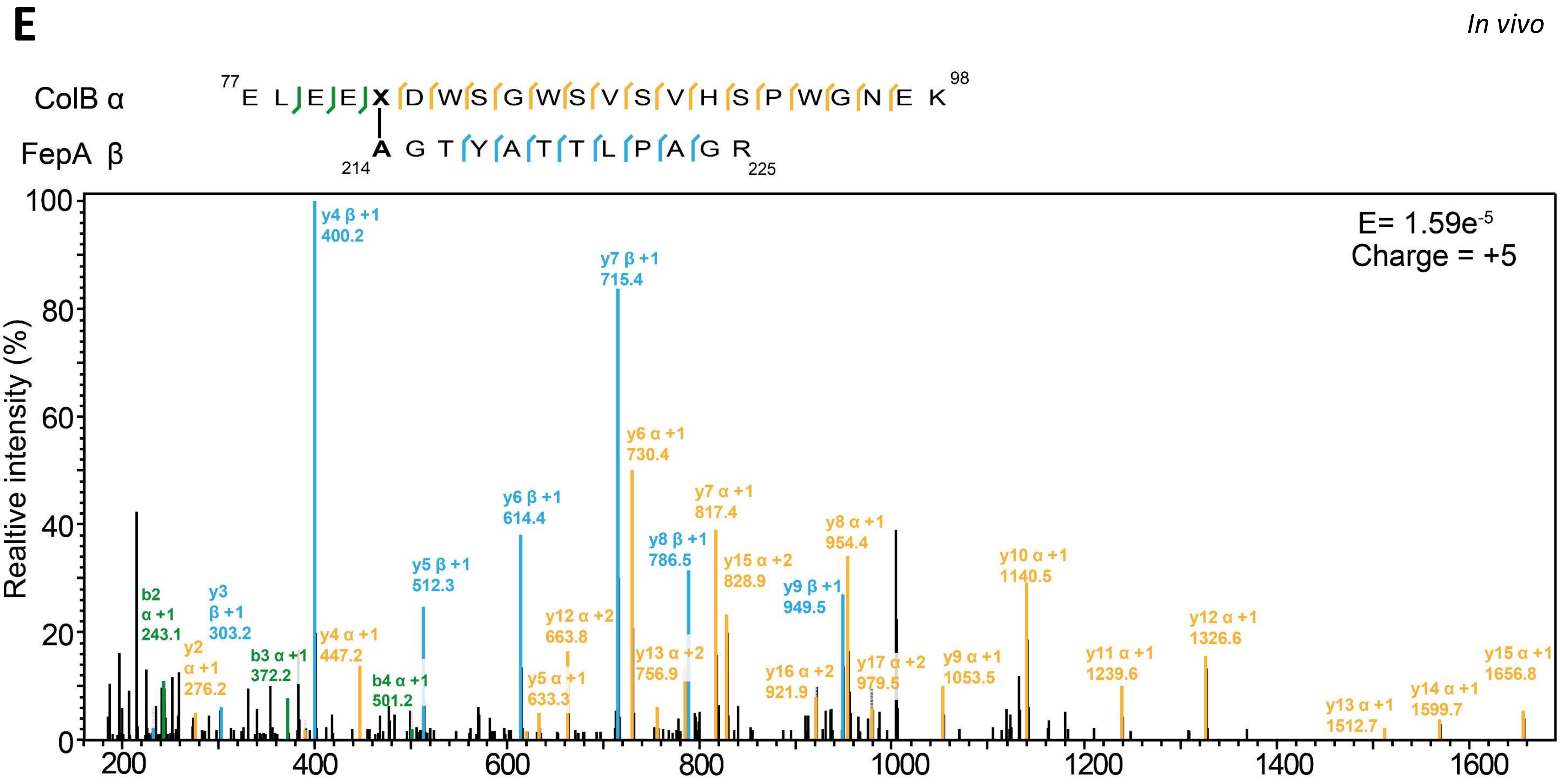

In vivo
ColB α
77 E L E E X D W S G W S V S V H S P W G N E K 98
FepA β
214 A G T Y A T T L P A G R 225
E= 1.59e-5
Charge = +5
Realtive intensity (%)
y4 β +1 400.2
y7 β +1 715.4
y6 α +1 730.4
y6 β +1 614.4
y7 α +1 817.4
y8 α +1 954.4
y8 β +1 786.5
y15 α +2 828.9
y5 β +1 512.3
y9 β +1 949.5
y10 α +1 1140.5
b2 α +1 243.1
y3 β +1 303.2
y12 α +2 663.8
y12 α +1 1326.6
y4 α +1 447.2
y2 α +1 276.2
b3 α +1 372.2
y13 α +2 756.9
y16 α +2 921.9
y17 α +2 979.5
y9 α +1 1053.5
y11 α +1 1239.6
y15 α +1 1656.8
b4 α +1 501.2
y5 α +1 633.3
y13 α +1 1512.7
y14 α +1 1599.7
