# Supplementary material for: Colicin-Mediated Transport of DNA through the Iron Transporter FepA
Source: mBio. 2021 Sep 21;12(5):e01787-21. doi: 10.1128/mBio.01787-21 (PMC8546555; doi:10.1128/mBio.01787-21)
Supplement: FIG S4 [file mbio.01787-21-sf004.pdf]

A

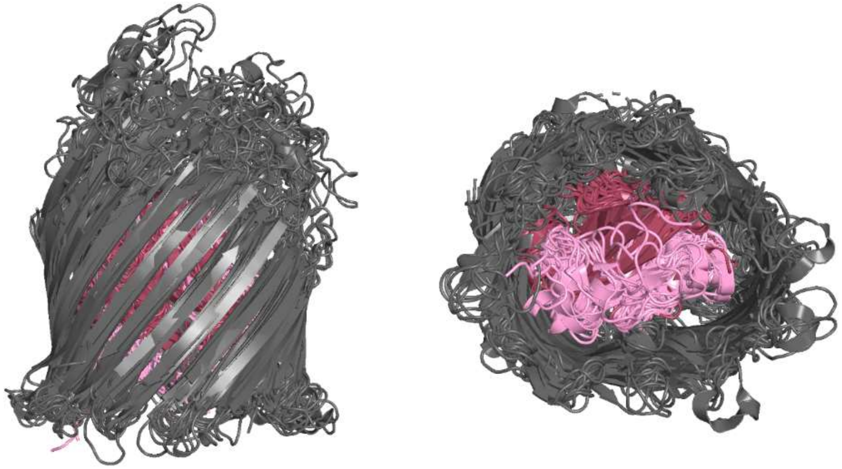

PDB	RMSD	Protein	Description	Origin
1FEP	0	FepA	Siderophore transporter	Escherichia coli
5MZS	1.23	PfepA	Siderophore transporter	Pseudomonas aeruginosa
5FR8	1.96	PirA	Siderophore transporter	Acinetobacter menangities
1QJQ	2.57	FhuA	Ferric hydroxamate receptor	Escherichia coli
4EPA	3.14	FyuA	Ferric yersiniabactin uptake receptor	Yersinia pestis
1NQH	3.19	BtuB	B12 Transporter	Escherichia coli
1PO0	3.26	FecA	Ferric citrate transporter in complex with iron-free citrate	Escherichia coli
2HDI	3.41	Cir	Colicin I receptor Cir in complex with RBD of Colicin Ia	Escherichia coli
3FHH	3.41	ShuA	Heme/Hemoglobin outer membrane transporter	Shigella dysenteriae
4AIP	3.48	FrpB	Iron transporter	Neisseria meningitdis
4RDT	3.62	ZnuD	Zn-transporter	Neisseria meningitdis
1XKW	3.85	FptA	Pyochelin outer membrane receptor	Pseudomonas aeruginosa
2IAH	3.93	FpvA	Ferripyoverdine receptor bound to substrate	Pseudomonas aeruginosa

B

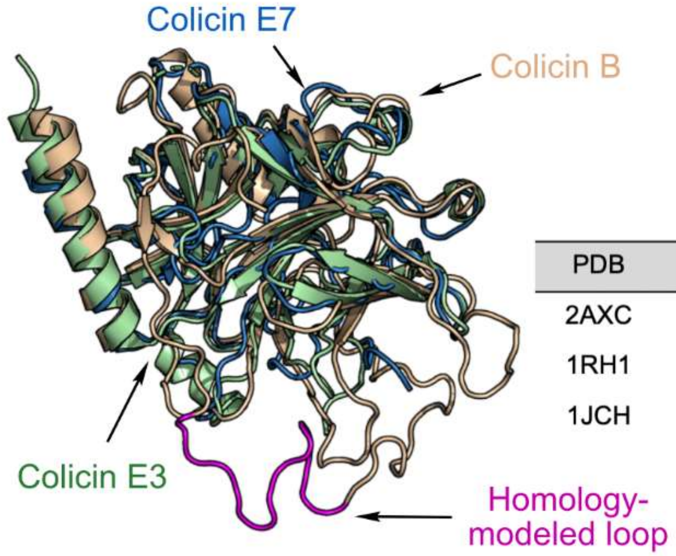

PDB	RMSD	Protein	Description	Origin
2AXC	0	Colicin E7	N'-terminal translocation domain	Escherichia coli
1RH1	2.38	Colicin B	N'-terminal translocation domain	Escherichia coli
1JCH	9.42	Colicin E3	N'-terminal translocation domain	Escherichia coli
