# Supplementary material for: Colicin-Mediated Transport of DNA through the Iron Transporter FepA
Source: mBio. 2021 Sep 21;12(5):e01787-21. doi: 10.1128/mBio.01787-21 (PMC8546555; doi:10.1128/mBio.01787-21)

**Supplementary computational Methods**

**PDB Curation and Minimization**

We acquire the crystal structures from the PDB (Colicin B, PDB ID: 1RH1 and enterobactin siderophore receptor FepA, 1FEP). We remove additional water and heteroatoms and clean the PDB structure of all the HETATM and non-canonical amino acids.

$ grep ATOM 1fep.pdb > 1fep_clean.pdb

$ grep ATOM 1rh1.pdb > 1rh1_clean.pdb

To construct the missing residues, we pass the amino-acid FASTA sequence of the monomer chains with their respectively template PDB structures on SWISS MODELLER, and build models for computational modeling. Before initiating the docking pipeline, we alleviate atomic clashes and optimize the structure with Rosetta Relax.

$ relax.linuxgccrelease

-in:file:s <PDB>

-relax:thorough

-relax:constrain_relax_to_start_coords

-nstruct 1

**Global Docking**

To determine putative local binding sites, we perform global docking with the Rosetta ReplicaDock2 protocol. The ReplicaDock protocol performs temperature replica exchange MC simulations that allows multiple replicas to communicate with each other and in turn improve the sampling of the energy landscape. We perform global docking in a low-resolution stage while restricting motion to only rigid body moves to better explore the protein energy landscape. To perform global docking, we initiate 8 trajectories of the docking simulation, each trajectory spanning over three temperature replicas run for 2 x 10^6^ MC steps. Inverse temperatures for the replicas are set to β, of 1.5^-1^ kcal^-1^.mol, 3^-1^ kcal^-1^.mol and 5^-1^ kcal^-1^.mol, and replica exchange swaps are performed every 1000 MC steps. The evaluation is based on interface energies calculated with a six-dimensional, residue-pair transform Motif Dock Score.

**Ensemble Generation methods**

A pre-generated ensemble effectively determines the ability of the conformer-selection approaches to select the most promising backbone conformations that can form a thermodynamically feasible complex structure. We use three methods to sample diverse backbone structures.

**ReplicaDock 2.0 (Induced-fit moves)**

We built and benchmarked a new method incorporating temperature and Hamiltonian REMC along with induced-fit motions in docking. In this local docking approach, we capture backbone motions of putative interface residues on-the-fly while docking. We perform the search on 8 trajectories all initiated at randomly oriented local binding sites, and each trajectory spans over three replicas run for 2 x 10^5^ MC steps. We set the temperatures, 1/β, of 1.5 kcal.mol^-1^, 3 kcal.mol^-1^ and 5 kcal.mol^-1^ for the low, medium and high temperatures respectively and replica exchange swaps are attempted every 1000 MC steps. We perform an all-atom refinement over the generated models and the top scoring 50 decoys are seeded into the ensemble. More details on how to utilize the protocol will be incorporated in our future work.

**Relax**

We utilize the Rosetta FastRelax protocol to sample backbone conformations of the monomers in isolation. The relax protocol is an equilibration protocol that performs side-chain packing, all-atom refinement and optimization in torsional space. For each monomer, we generate 25 decoys to seed the ensemble.

$ relax.linuxgccrelease

-in:file:s <PDB>

-relax:fast

-nstruct 25

**Backrub**

To perform backbone flexing of the protein fragments, we use the Rosetta Backrub protocol that samples orientations about an axis defined by the pivot atoms i.e. start and end atoms of the fragment. This is performed in isolation for the ligand and receptor chains. We seed the ensemble with 25 backrub outputs.

$ backrub.linuxgccrelease

-in:file:s <PDB>

-backrub:mc_kt 0.6

-backrub:ntrials 20000

-nstruct 25

**Docking simulations**

Upon generating the ensemble, we follow the pre-packing and docking steps of the RosettaDock 4.0 protocol to perform docking. The details are elaborated in prior work by Marze et al. The command line options for this case are as follows:

**Prepacking**

**Docking**

$ docking_protocol.linuxgccrelease

-in:file:s <PDB>

-in:file:native <Reference PDB>
 # this is for estimation of I_rmsd and L_rmsd metrics

-partners A_B

-docking_local_refine

-ex1 -ex2aro -rebuild_disulf true

-detect_disulf true

-nstruct 1

$ docking_prepack_protocol.linuxgccrelease

-in:file:s <PDB>

-ensemble1 <Receptor Ensemble List>

-ensemble2 <Ligand Ensemble List>

-partners A_B

-ex1 -ex2aro -rebuild_disulf true

-detect_disuf true

-nstruct 1

**All-atom Docking Refinement**

$ docking_protocol.linuxgccrelease

-in:file:s <PDB>

-in:file:native <Reference PDB>
 # this is for estimation of I_rmsd and L_rmsd metrics

-partners A_B

-docking_local_refine

-ex1 -ex2aro -rebuild_disulf true

-detect_disulf true

-nstruct 1

**Rosetta FloppyTail**

We adapt the Rosetta FloppyTail protocol elaborately described in Kleiger et. Al^57^, Crawley et al. and Zhang et al. To summarize, FloppyTail samples larger conformational changes by perturbing the backbone dihedral angles via small and/or shear moves in low-resolution and all-atom stages. Each FloppyTail cycle undergoes gradient-based optimization and outputs a ‘refined’ decoy.

$ FloppyTail.linuxgccrelease

-in:file:s <PDB>

-ex1 -ex2 -use_input_sc

# to avoid changing the AA identities during packing

-packing:repack_only

-run:min_type dfpmin_armijo_nonmonotone

# Defines the residues to move

-in:file:movemap floppy_movemap

# MC sampling options

-FloppyTail:shear_on 0.25

-FloppyTail:publication false

-FloppyTail:refine_repack_cycles 10

-FloppyTail:perturb_cycles 5000

-FloppyTail:refine_cycles 1000

# Use constraints to direct sampling in low and high resolution modes

-constraints:cst_fa_file crosslink.cst

-constraints:cst_file crosslink.cst

-constraints:cst_weight 1.0

-constraints:cst_fa_weight 1.0

# Set the root at C-term because we want the N-term to be flexible

-C_root true

-force_linear_fold_tree

-nstruct 5000

-out:pdb_gz

The movemap file defines the backbone degrees of freedom that are set for the residues. An example of a sample file that moves the residues (residue numbering follows Rosetta Standard numbering style i.e. they are continuously number from 1 based on their appearance in the PDB file).

RESIDUE * CHI # defaults packing

JUMP * NO # No movement of subunits with respect to each other

RESIDUE 1 76 BBCHI

We have also defined constraints based on the experimental crosslinking data and these constraints effectively direct the sampling of backbone degrees of freedom in relevant search space. We use harmonic constraints and the constraint file is as follows:

# Constraint files also follow Rosetta Numbering

AtomPair CA 48A CA 10B HARMONIC 6.0 0.25

AtomPair CA 204A CA 72B HARMONIC 6.0 0.25

AtomPair CA 629A CA 193B HARMONIC 6.0 0.25

AtomPair CA 632A CA 196B HARMONIC 6.0 0.25

AtomPair CA 642A CA 46B HARMONIC 6.0 0.25

**Computational Workflow for ColB-FepA complex prediction**

1. Workflow for identifying semi-rigid encounter complex (EC)


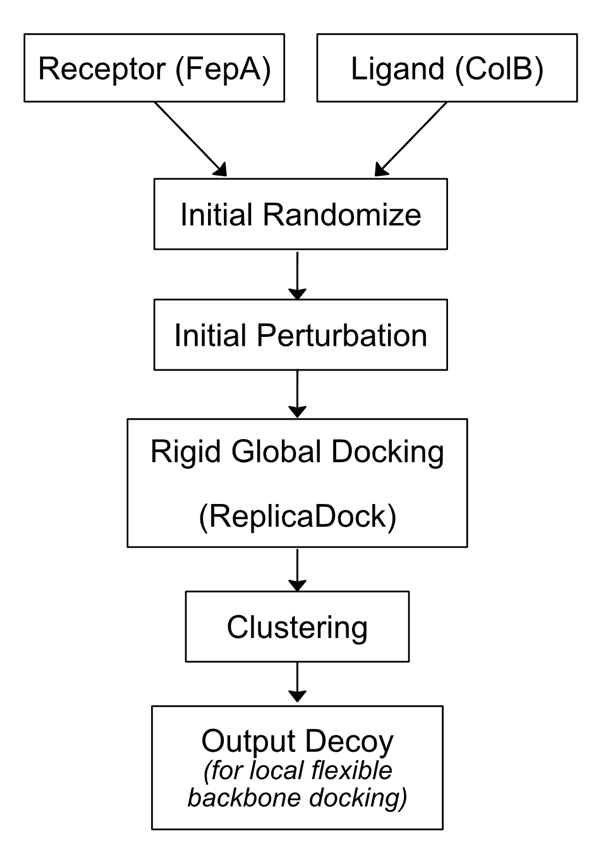


1. Workflow for modeling the flexible encounter complex (SC) between FepA-ColB.


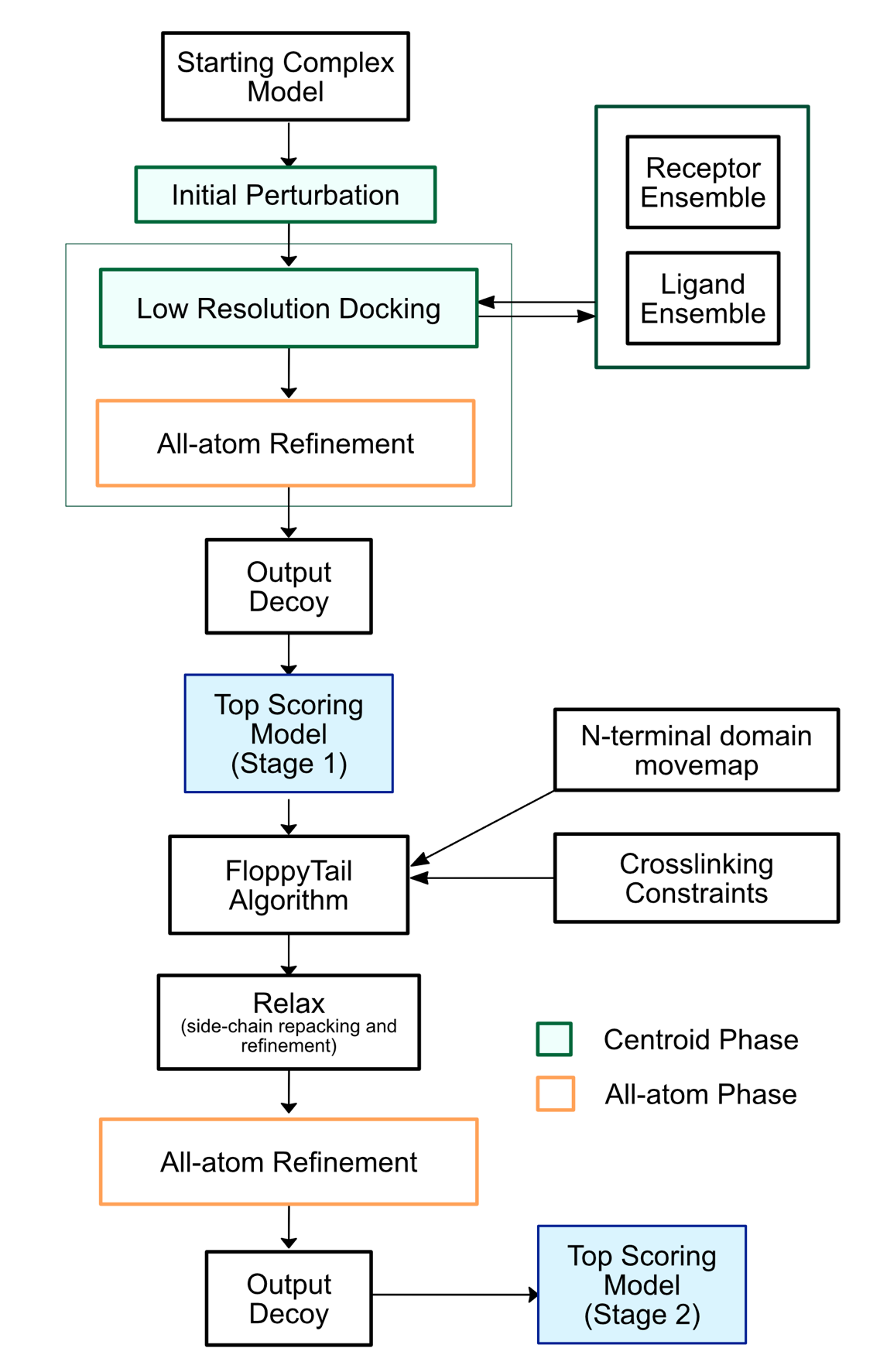


1. Workflow for modeling the complexes in the translocation pathway


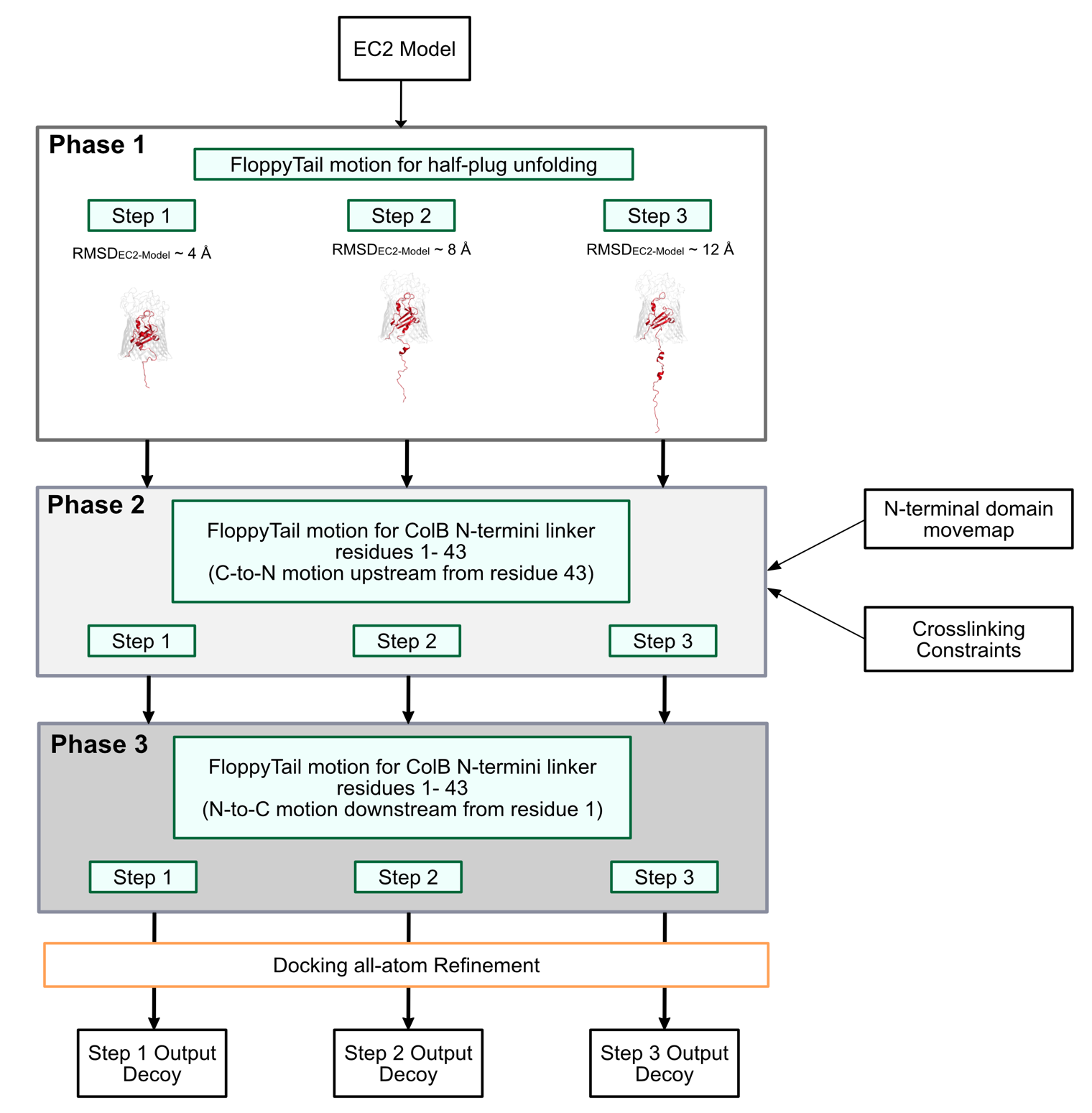

Supplement: TEXT S1 [file mbio.01787-21-s0001.docx]
